# Supplementary material for: Briarenones A‒C, New Briarellin Diterpenoids from the Gorgonian Briareum violaceum
Source: Mar Drugs. 2019 Feb 17;17(2):120. doi: 10.3390/md17020120 (PMC6409734; doi:10.3390/md17020120)
Supplement: Supplementary file 1 [file marinedrugs-17-00120-s001.pdf]

Briarenones A-C, New Briarellin diterpenoids from the gorgonian *Briareum violaceum*

Cheng Yang [1], Atallah F. Ahmed [2,3], Raha S. Orfali [2], Chang-Feng Dai [4], and Jyh-Horng Sheu [1,5,6,7,*]

[1] Department of Marine Biotechnology and Resources, National Sun Yat-sen University, Kaohsiung 804, Taiwan; jack1991106@yahoo.com.tw (C.-C. P)
[2] Department of Pharmacognosy, College of Pharmacy, King Saud University, Riyadh 11451, Saudi Arabia; afahmed@ksu.edu.sa; rorfali@ksu.edu.sa (R.S.O.)
[3] Department of Pharmacognosy, Faculty of Pharmacy, Mansoura University, Mansoura 35516, Egypt
[4] Institute of Oceanography, National Taiwan University, Taipei 112, Taiwan; corallab@ntu.edu.tw
[5] Graduate Institute of Natural Products, Kaohsiung Medical University, Kaohsiung 807, Taiwan
[6] Frontier Center for Ocean Science and Technology, National Sun Yat-sen University, Kaohsiung 804, Taiwan
[7] Department of Medical Research, China Medical University Hospital, China Medical University, Taichung 404, Taiwan

* Correspondence: sheu@mail.nsysu.edu.tw

List of Supplementary material

No.	Content	Page
**Figure S1**	HRESIMS spectrum of **1**	S3
**Figure S2**	^{1}H NMR spectrum of **1** in $CDCl_3$ at 600 MHz	S4
**Figure S3**	^{1}H NMR spectrum of **1** in $CDCl_3$ (1.08~2.82 and 3.10~5.92 ppm) at 600 MHz	S5
**Figure S4**	^{13}C NMR spectrum of **1** in $CDCl_3$ at 150 MHz and DEPT spectra	S6
**Figure S5**	HSQC spectrum of **1** in $CDCl_3$	S7
**Figure S6**	^{1}H -^{1}H COSY spectrum of **1** in $CDCl_3$	S8
**Figure S7**	HMBC spectrum of **1** in $CDCl_3$	S9
**Figure S8**	ROESY spectrum of **1** in $CDCl_3$	S10
**Figure S9**	HRESIMS spectrum of **2**	S11
**Figure S10**	^{1}H NMR spectrum of **2** in $CDCl_3$ at **5**00 MHz	S12
**Figure S11**	^{1}H NMR spectrum of **2** in $CDCl_3$ (0.96~3.28 and 3.24~6.08ppm) at 500 MHz	S13
**Figure S12**	^{13}C NMR spectrum of **2** in $CDCl_3$ at 1**25** MHz	S14
**Figure S13**	DEPT spectra of **2** in $CDCl_3$	S15
**Figure S14**	HSQC spectrum of **2** in $CDCl_3$	S16
**Figure S15**	^{1}H -^{1}H COSY spectrum of **2** in $CDCl_3$	S17
**Figure S16**	HMBC spectrum of **2** in $CDCl_3$	S18
**Figure S17**	NOESY spectrum of **2** in $CDCl_3$	S19
**Figure S18**	HRESIMS spectrum of **3**	S20
**Figure S19**	^{1}H NMR spectrum of **3** in $CDCl_3$ at **5**00 MHz	S21
**Figure S20**	^{1}H NMR spectrum of **3** in $CDCl_3$ (0.82~3.04 and 3.38~ 6.03 ppm) at 500 MHz	S22
**Figure S21**	^{13}C NMR spectrum of **3** in $CDCl_3$ at 1**25** MHz	S23
**Figure S22**	DEPT spectra of **3** in $CDCl_3$	S24
**Figure S23**	HSQC spectrum of **3** in $CDCl_3$	S25
**Figure S24**	^{1}H -^{1}H COSY spectrum of **3** in $CDCl_3$	S26
**Figure S25**	HMBC spectrum of **3** in $CDCl_3$	S27
**Figure S26**	NOESY spectrum of **3** in $CDCl_3$	S28
**Table S1.**	Crystal data and structure refinement for **1**	S29
**Table S2**	Atomic coordinates (x 104) and equivalent isotropic displacement parameters (Å2x 103) for **1**	S30
**Table S3**	Bond lengths [Å] and angles [°] for **1**	S31
**Table S3**	Bond lengths [Å] and angles [°] for **1** (contd.)	S32

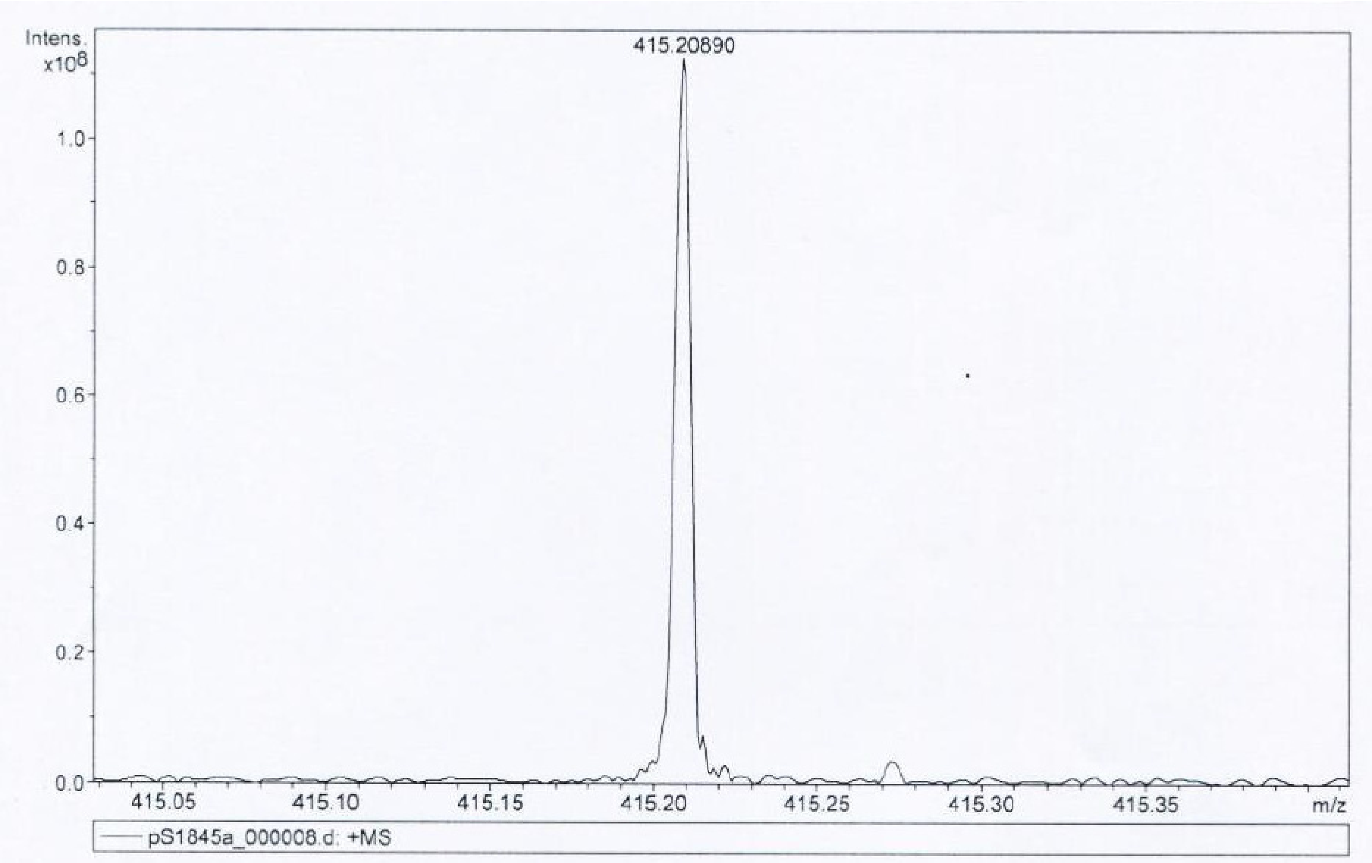

Meas. m/z	#	Formula	Score	m/z	err [mDa]	err [ppm]	mSigma	rdb	e⁻ Conf	N-Rule
415.20890	1	C 22 H 32 Na O 6	100.00	415.20911	0.21	0.51	10.6	6.5	even	ok

Figure S1. HRESIMS spectrum of **1**

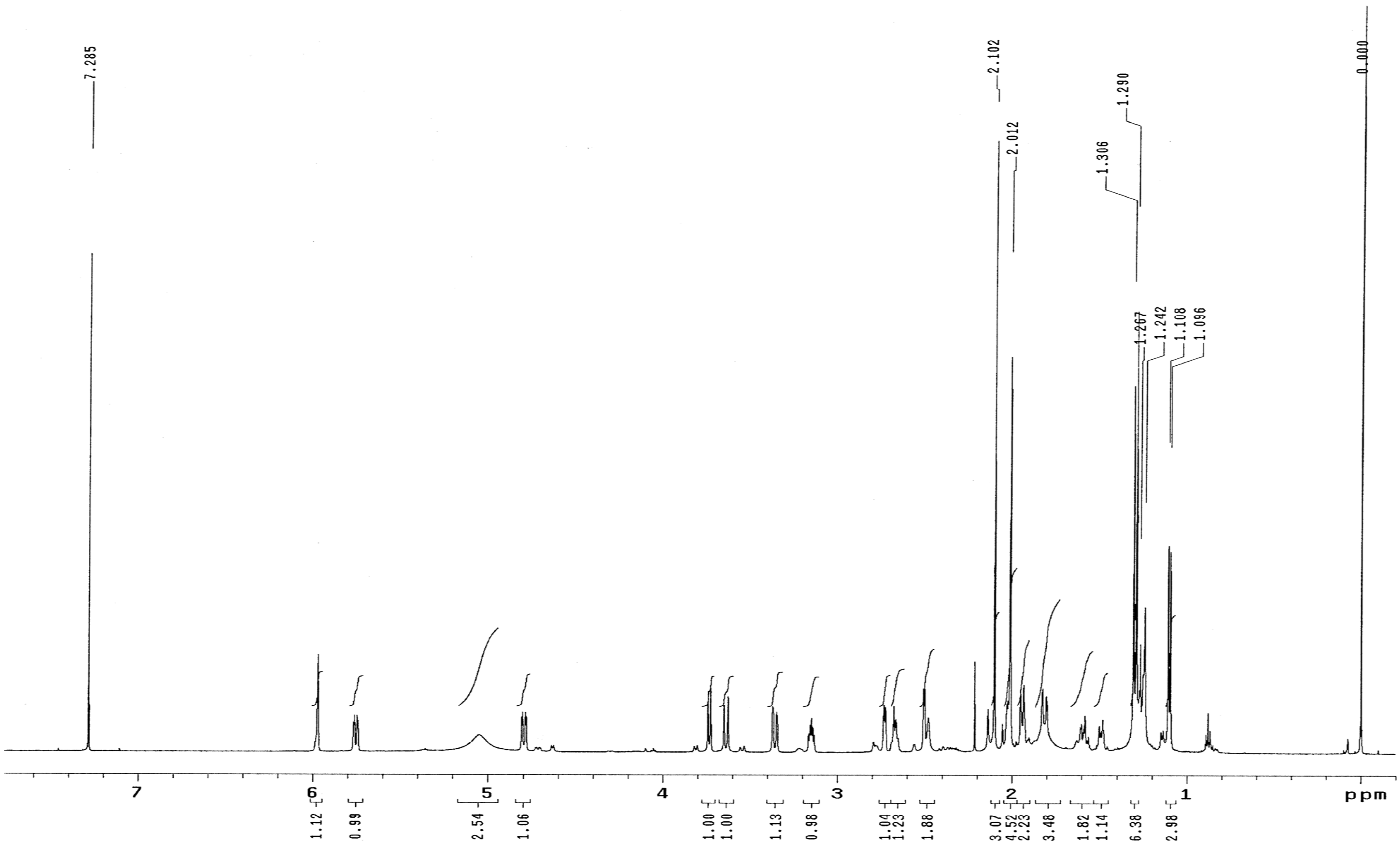

Figure S2. ^{1}H NMR spectrum of **1** in $CDCl_3$ at 600 MHz

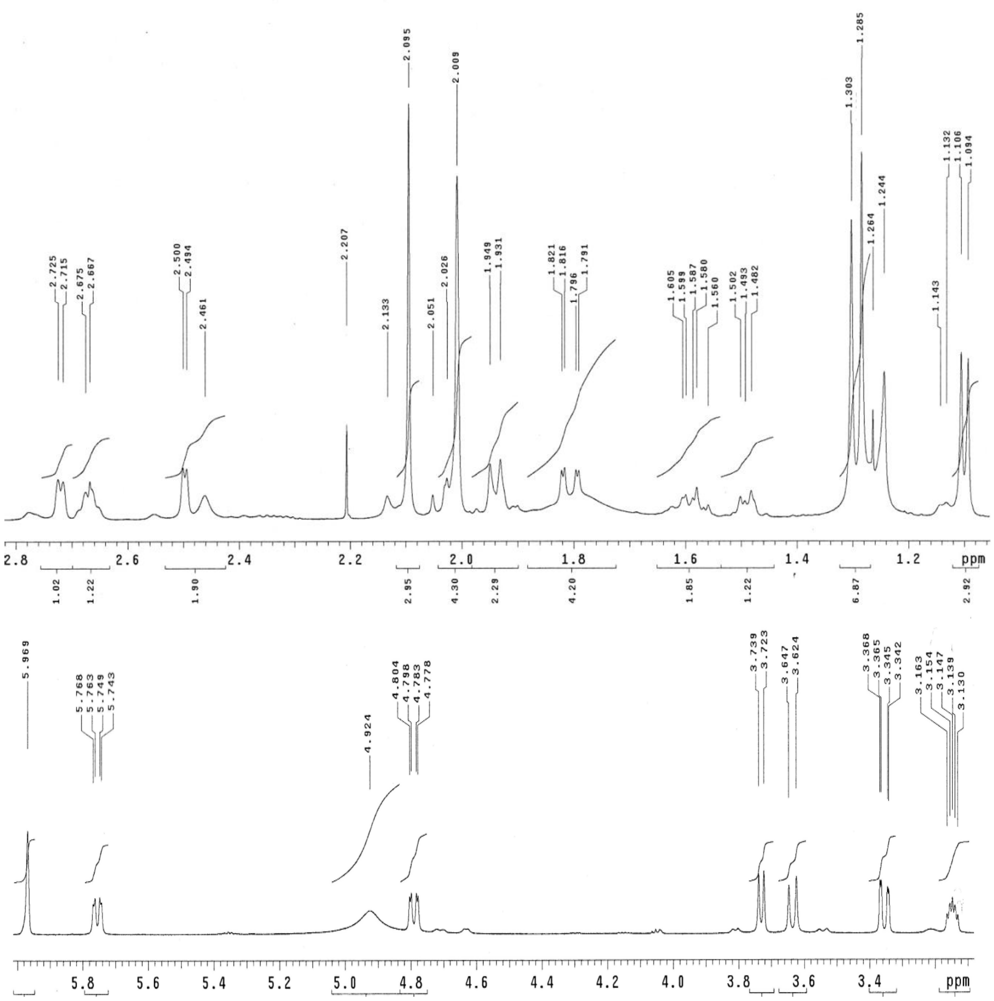

Figure S3. ^{1}H NMR spectrum of **1** in $CDCl_3$ (1.08~2.82 and 3.10~5.92 ppm) at 600 MHz

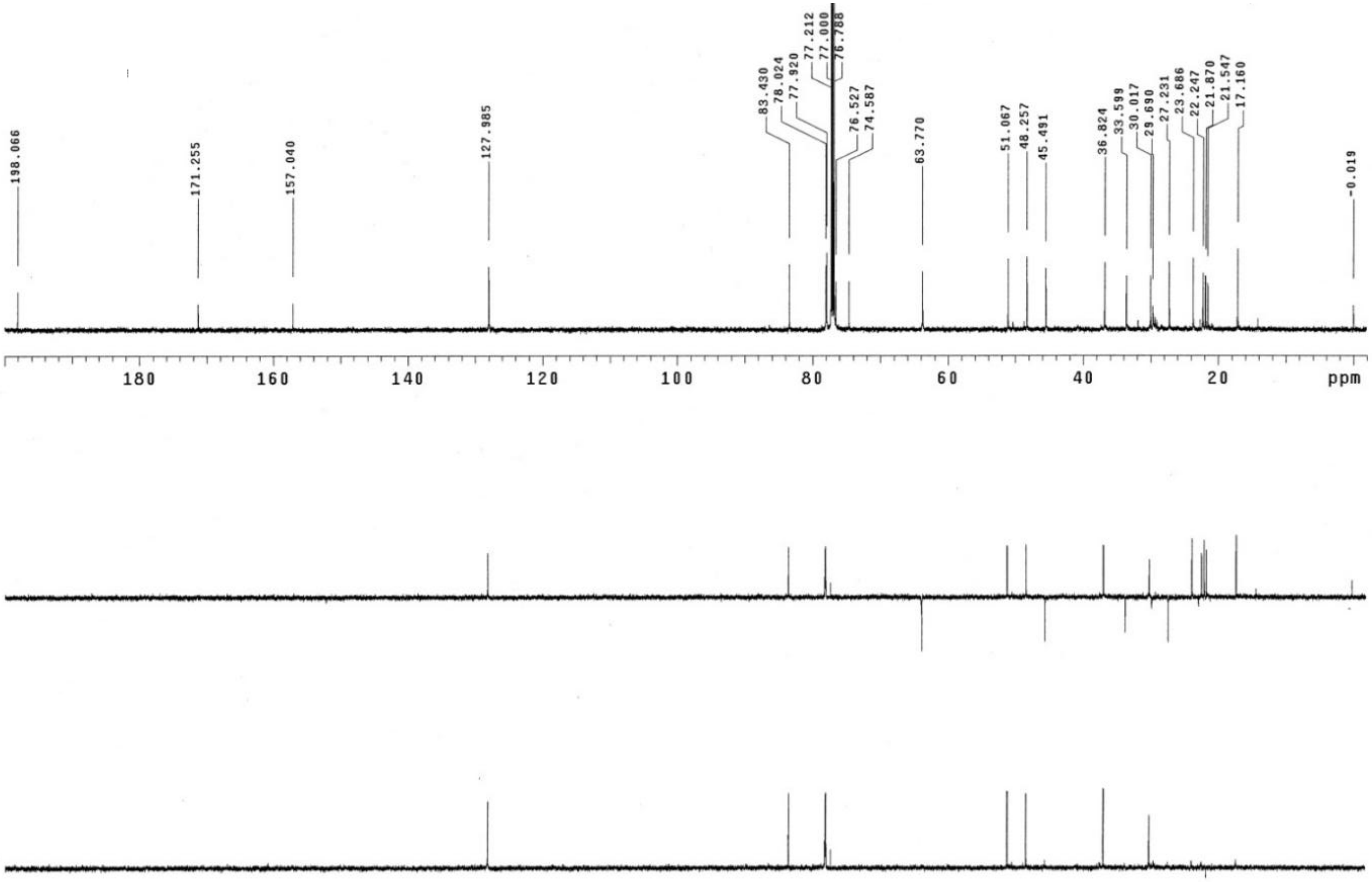

Figure S4. ^{13}C NMR spectrum of **1** in $CDCl_3$ at 150 MHz and DEPT spectra

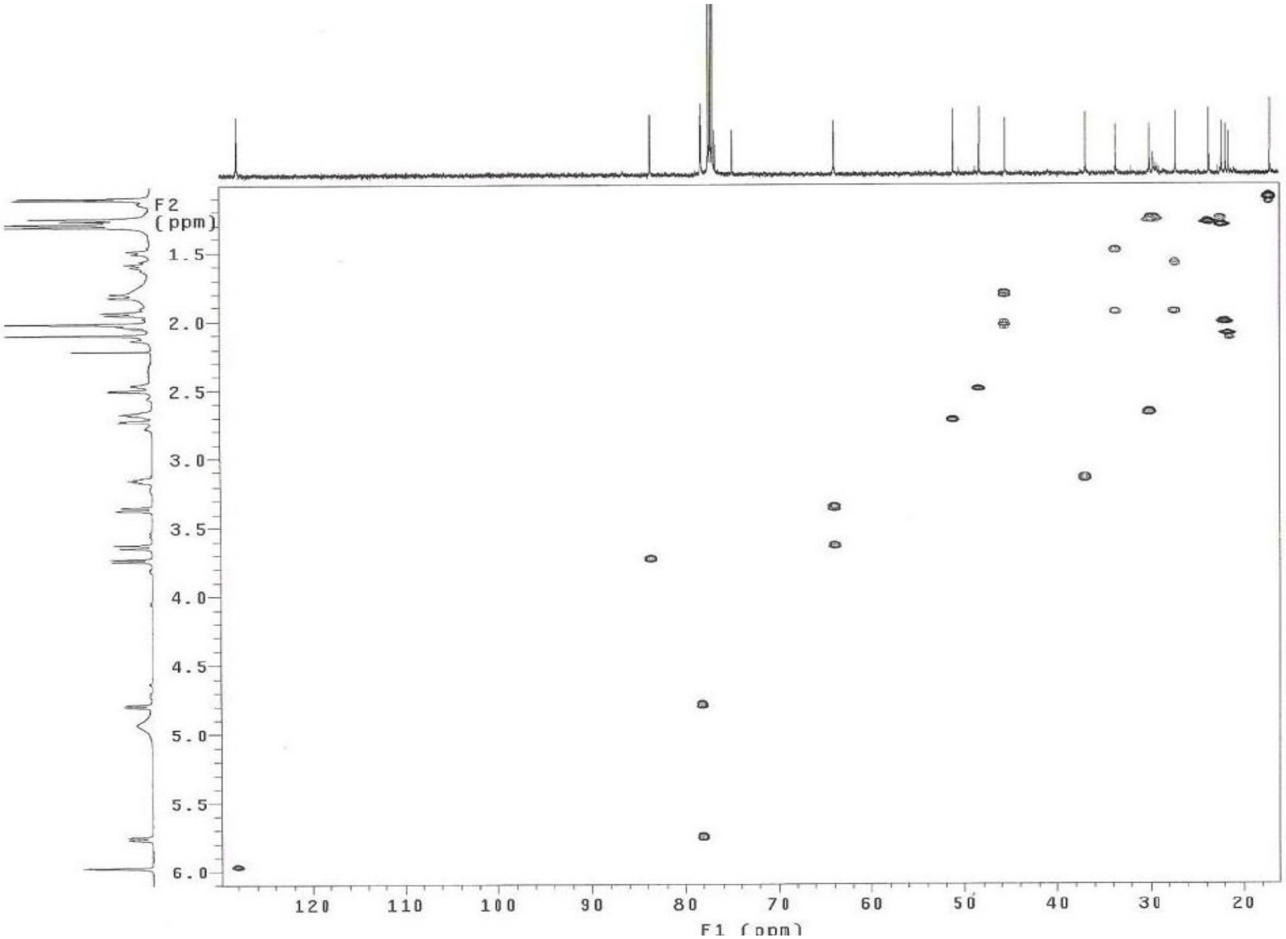

Figure S5. HSQC spectrum of **1** in $CDCl_3$

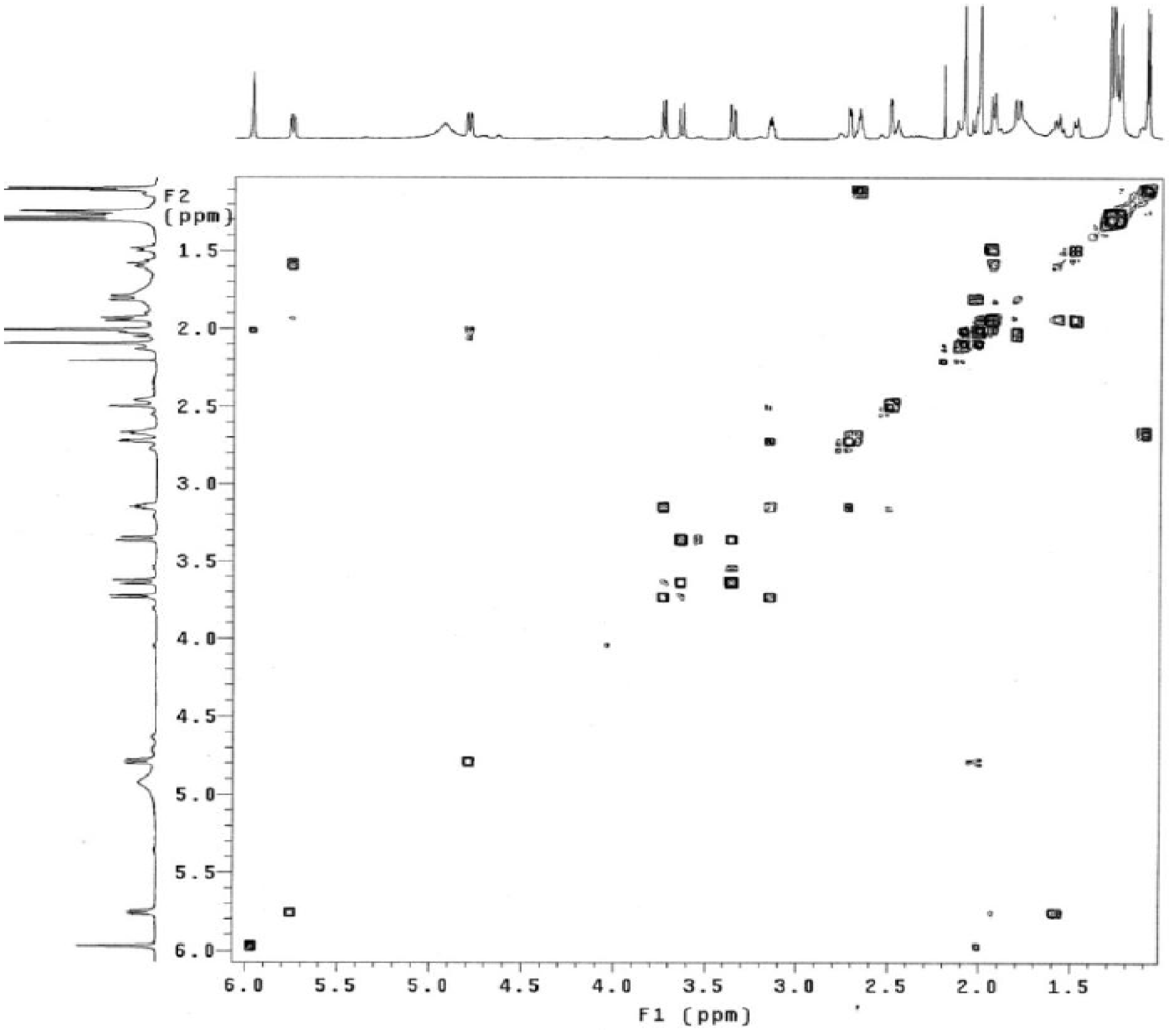

Figure S6. ^{1}H -^{1}H COSY spectrum of **1** in $CDCl_3$

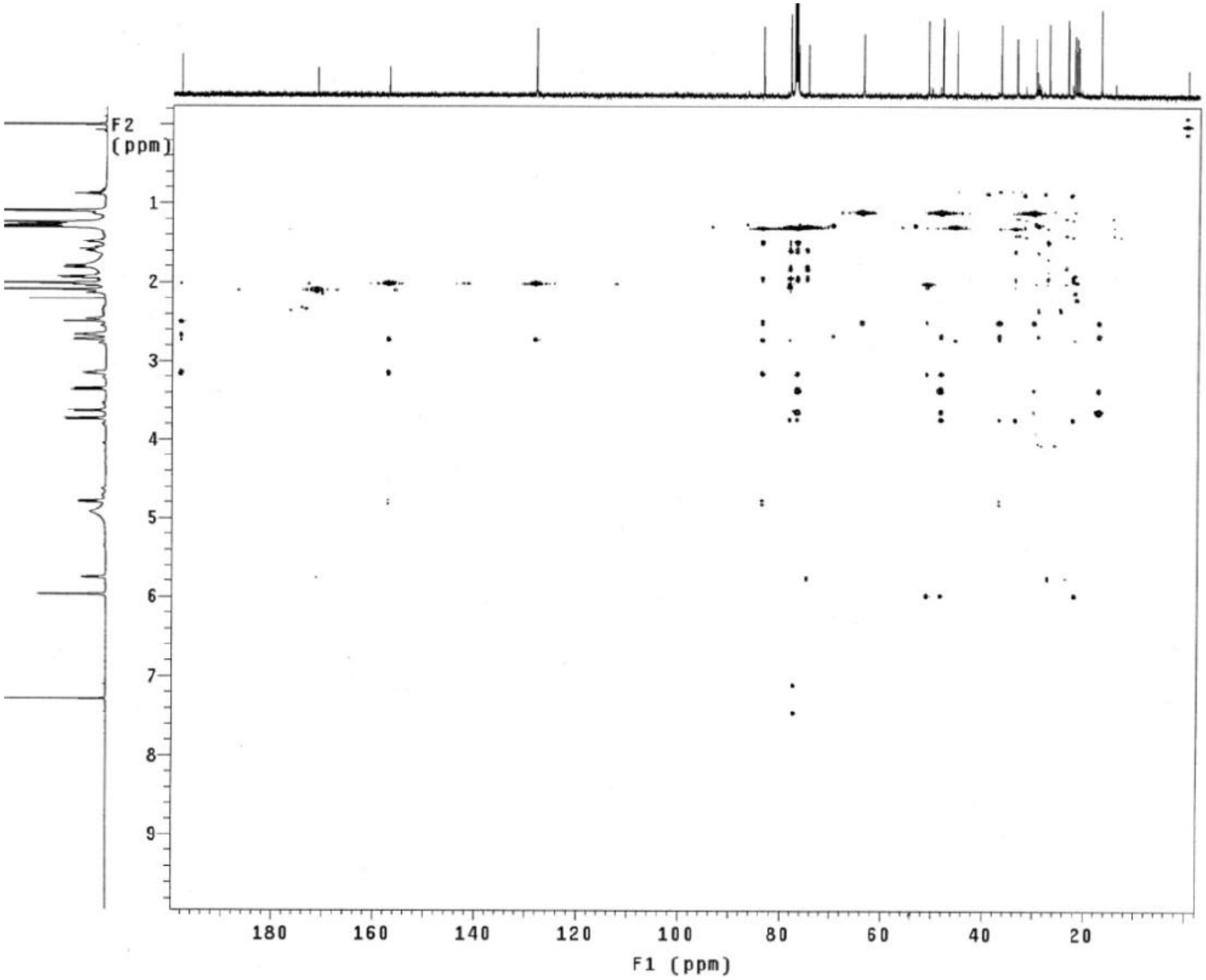

Figure S7. HMBC spectrum of **1** in $CDCl_3$

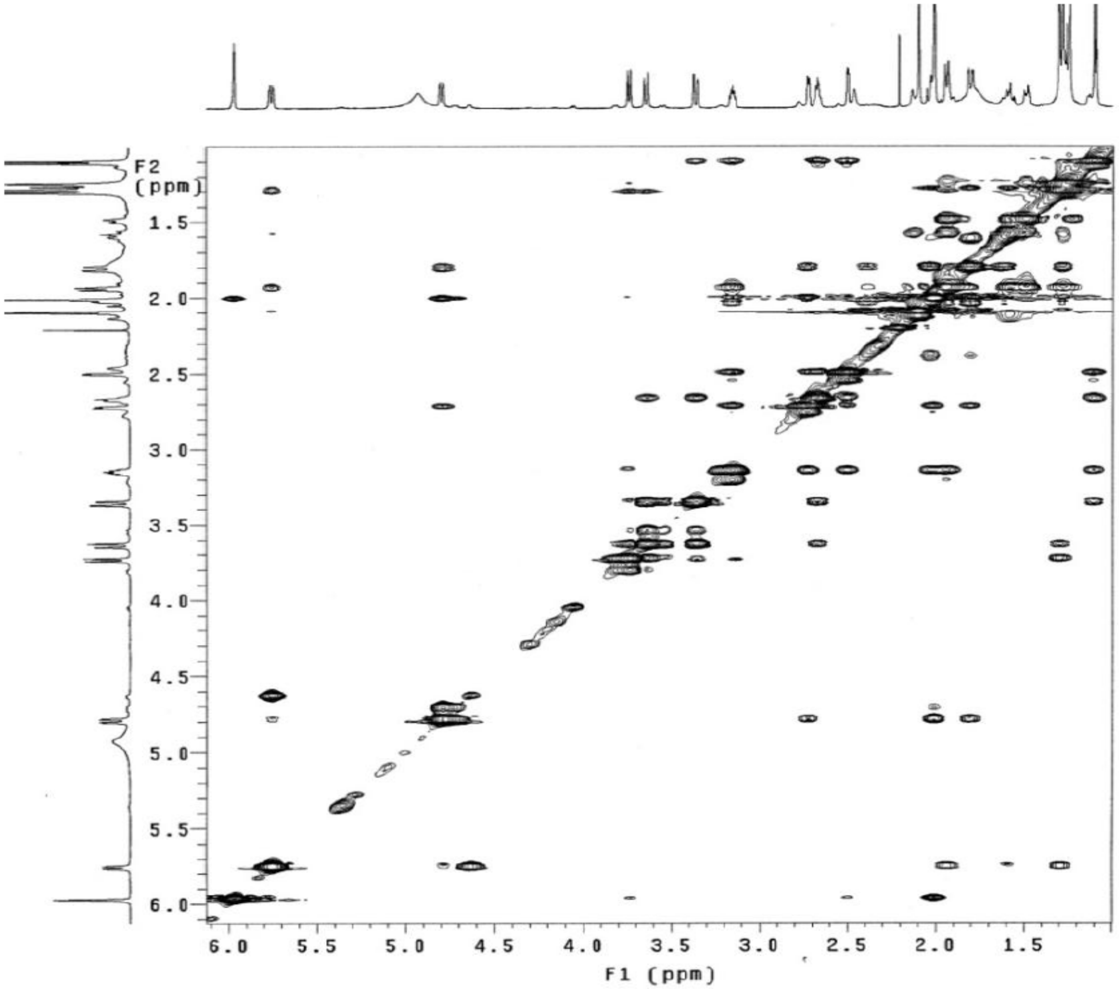

Figure S8. ROESY spectrum of **1** in $CDCl_3$

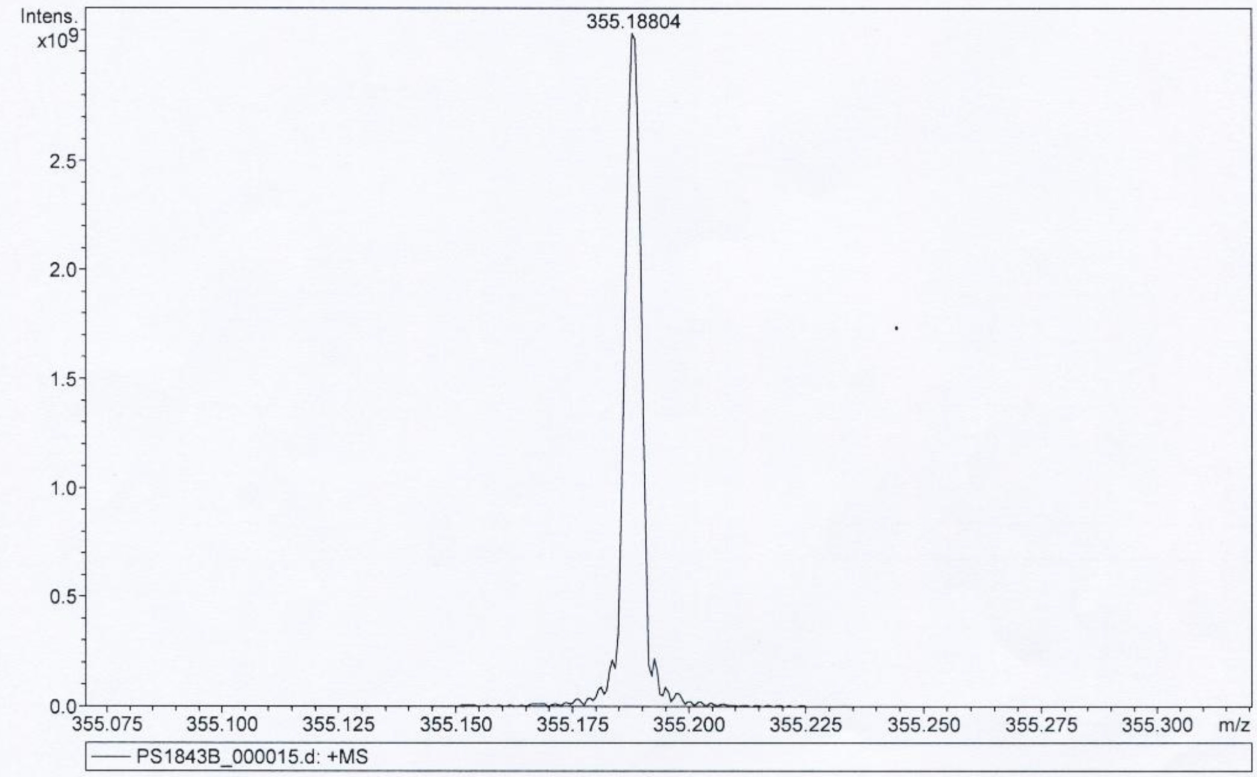

Meas. m/z	#	Formula	Score	m/z	err [mDa]	err [ppm]	mSigma	rdb	e⁻ Conf	N-Rule
355.18804	1	C 20 H 28 Na O 4	100.00	355.18798	-0.06	-0.18	13.2	6.5	even	ok

Figure S9. HRESIMS spectrum of **2**

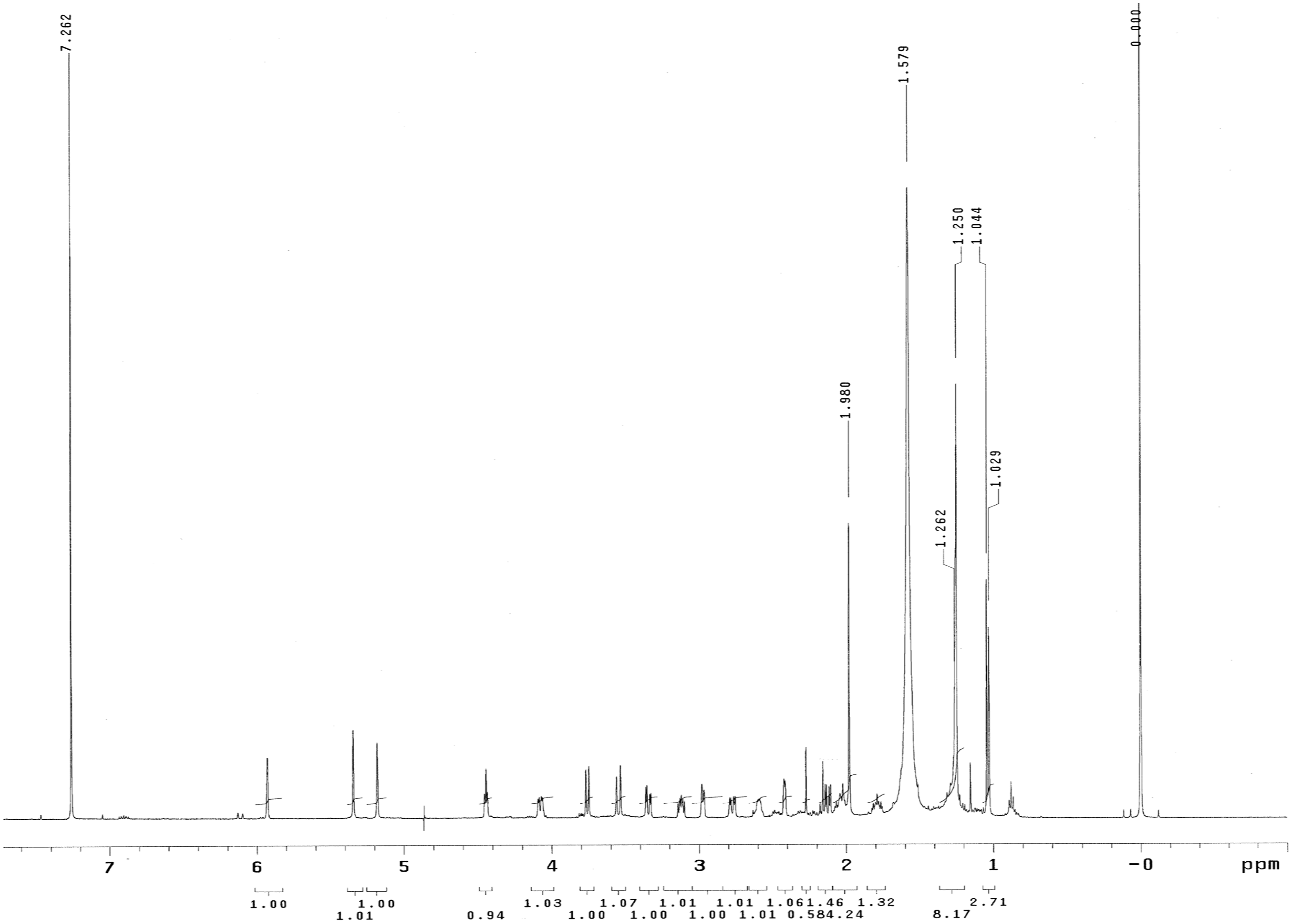

Figure S10. ^{1}H NMR spectrum of **2** in $CDCl_3$ at 500 MHz

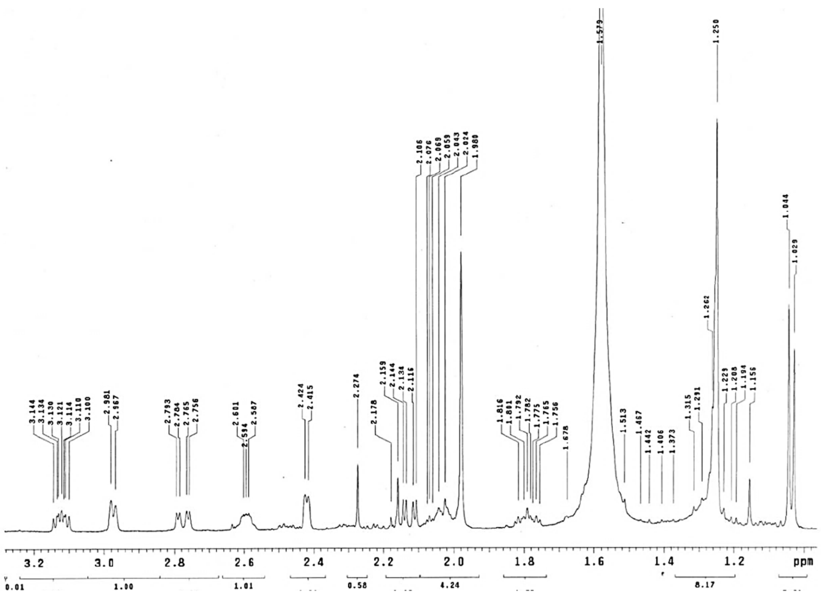

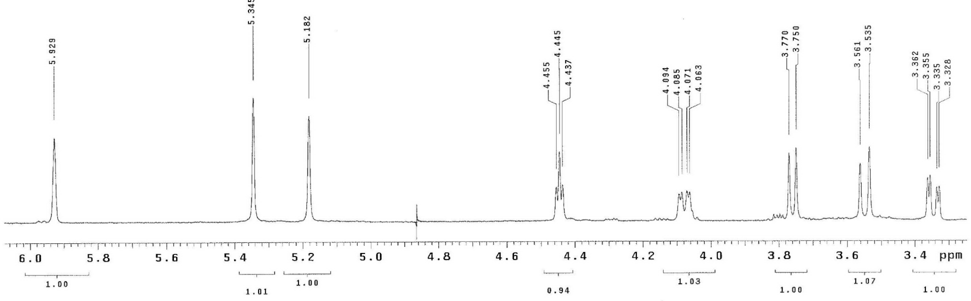

Figure S11 ^{1}H NMR spectrum of **2** in $CDCl_3$ (0.96~3.28 and 3.24~6.08ppm) at 500 MHz

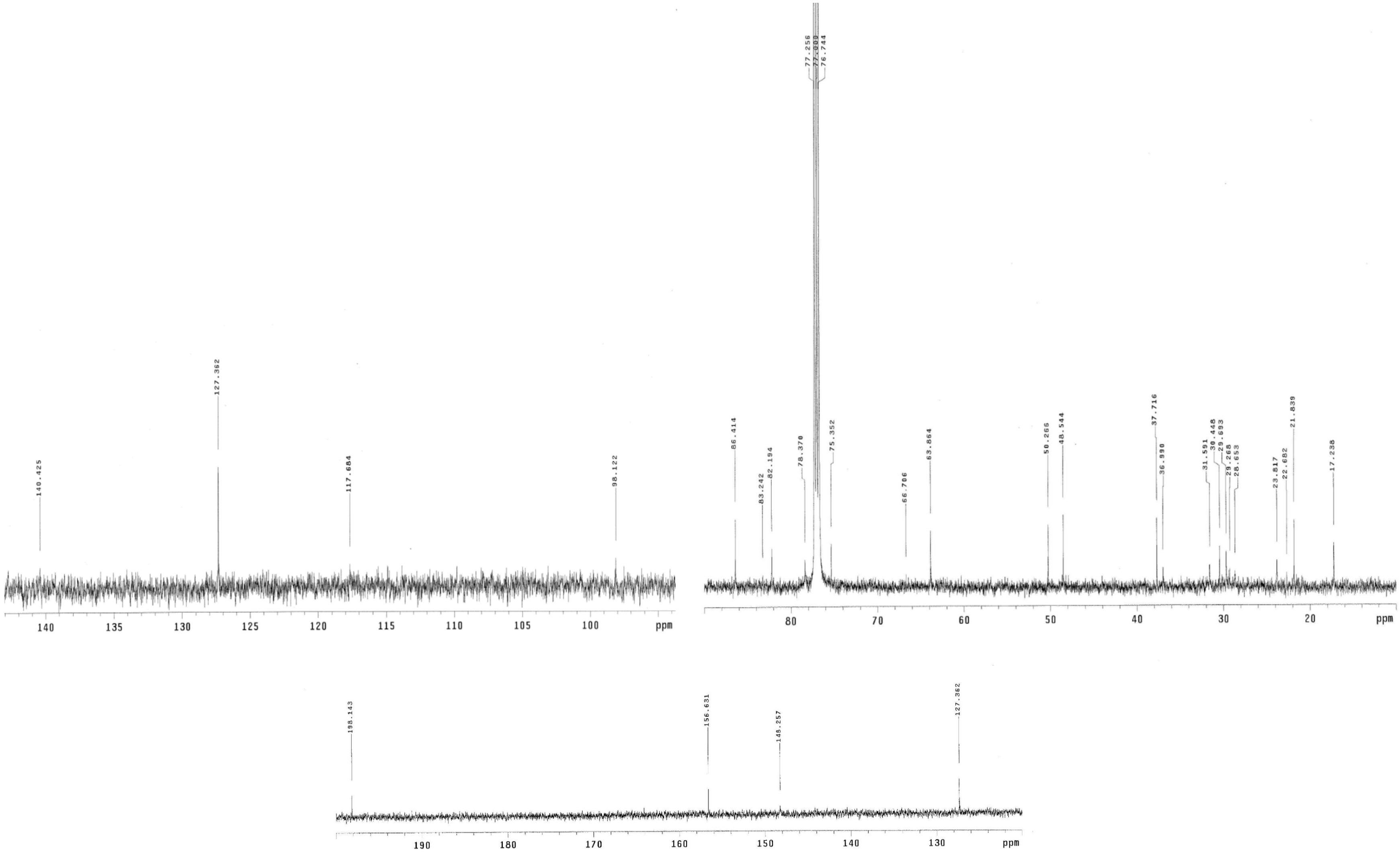

Figure S12. ^{13}C NMR spectrum of **2** in $CDCl_3$ at 125 MHz

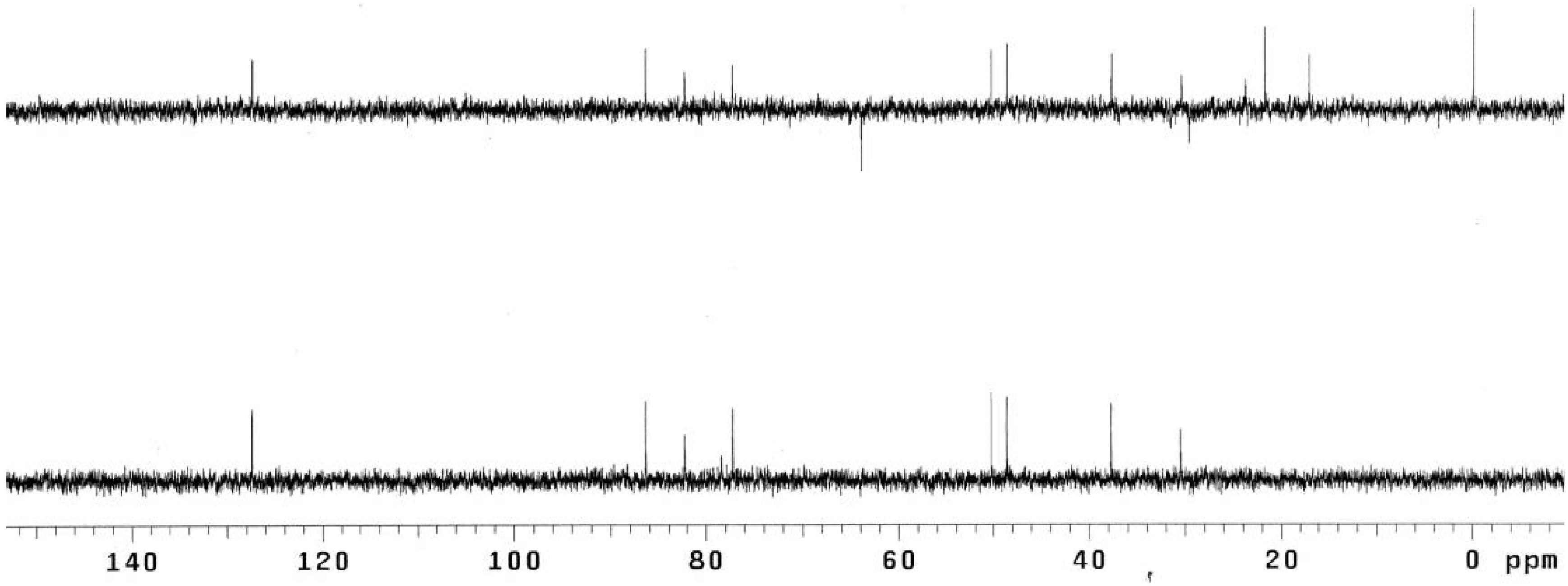

Figure S13. DEPT spectra of **2** in $CDCl_3$

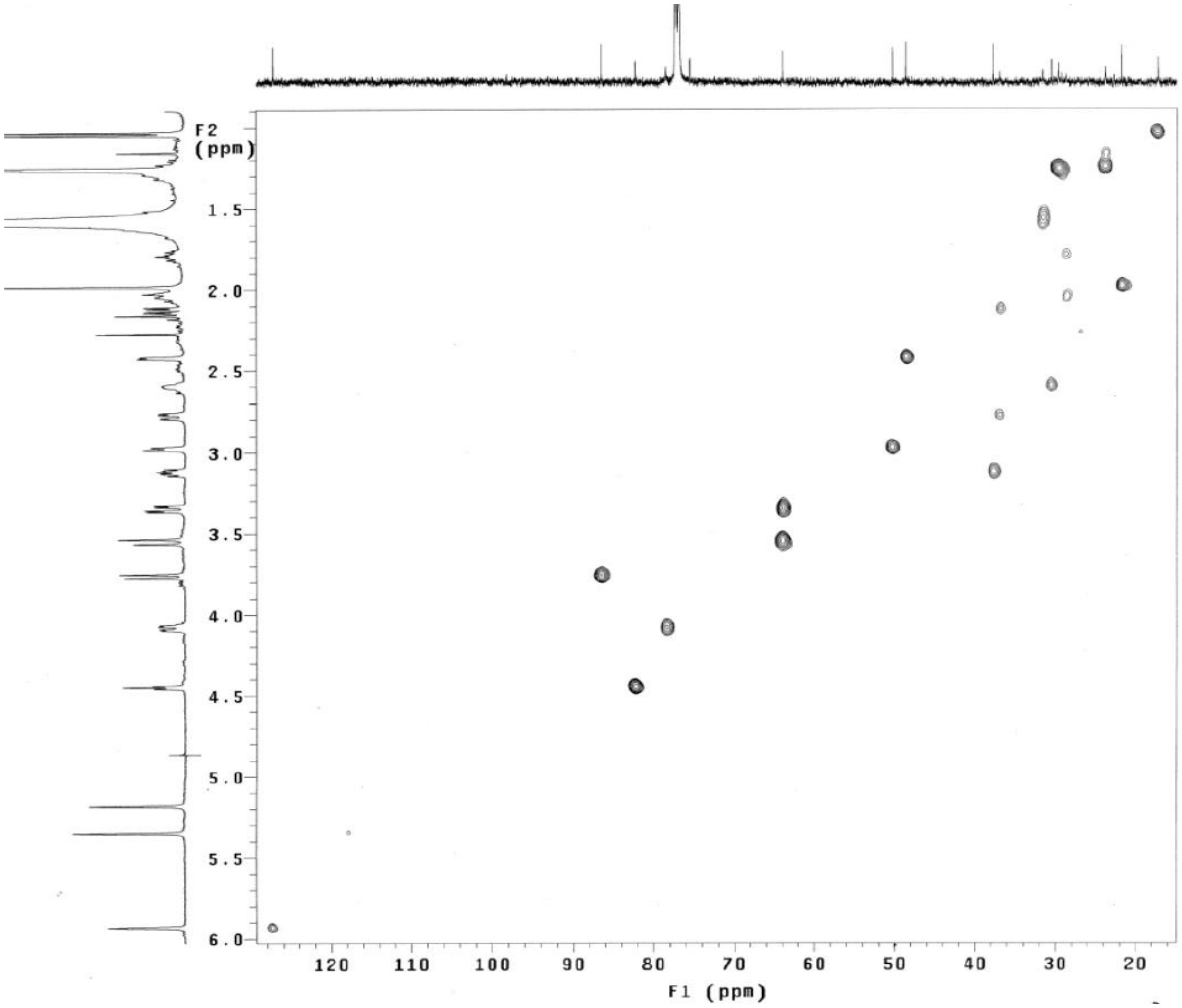

Figure S14. HSQC spectrum of **2** in $CDCl_3$

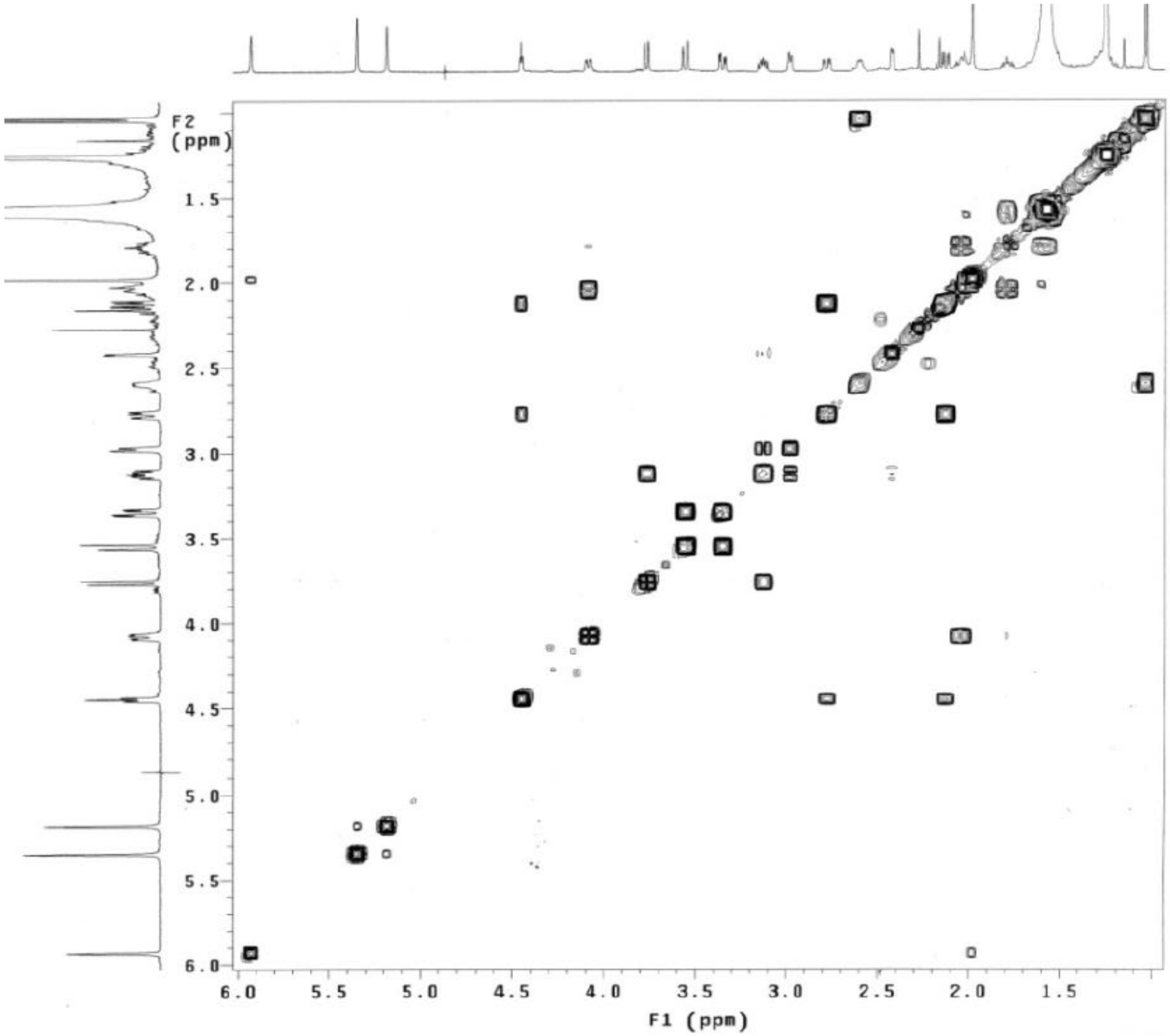

Figure S15. ^{1}H -^{1}H COSY spectrum of **2** in $CDCl_3$

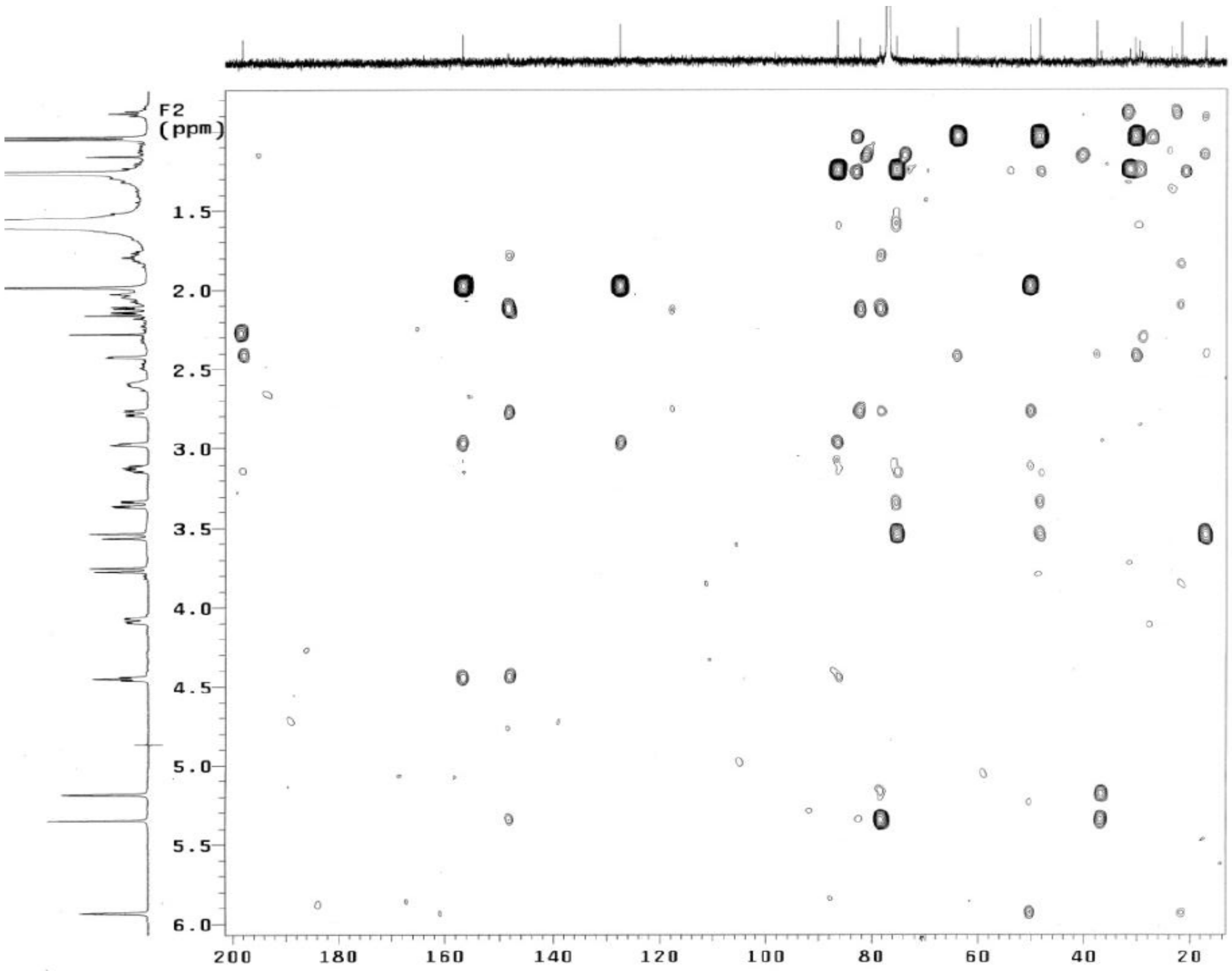

Figure S16. HSQC spectrum of **2** in $CDCl_3$

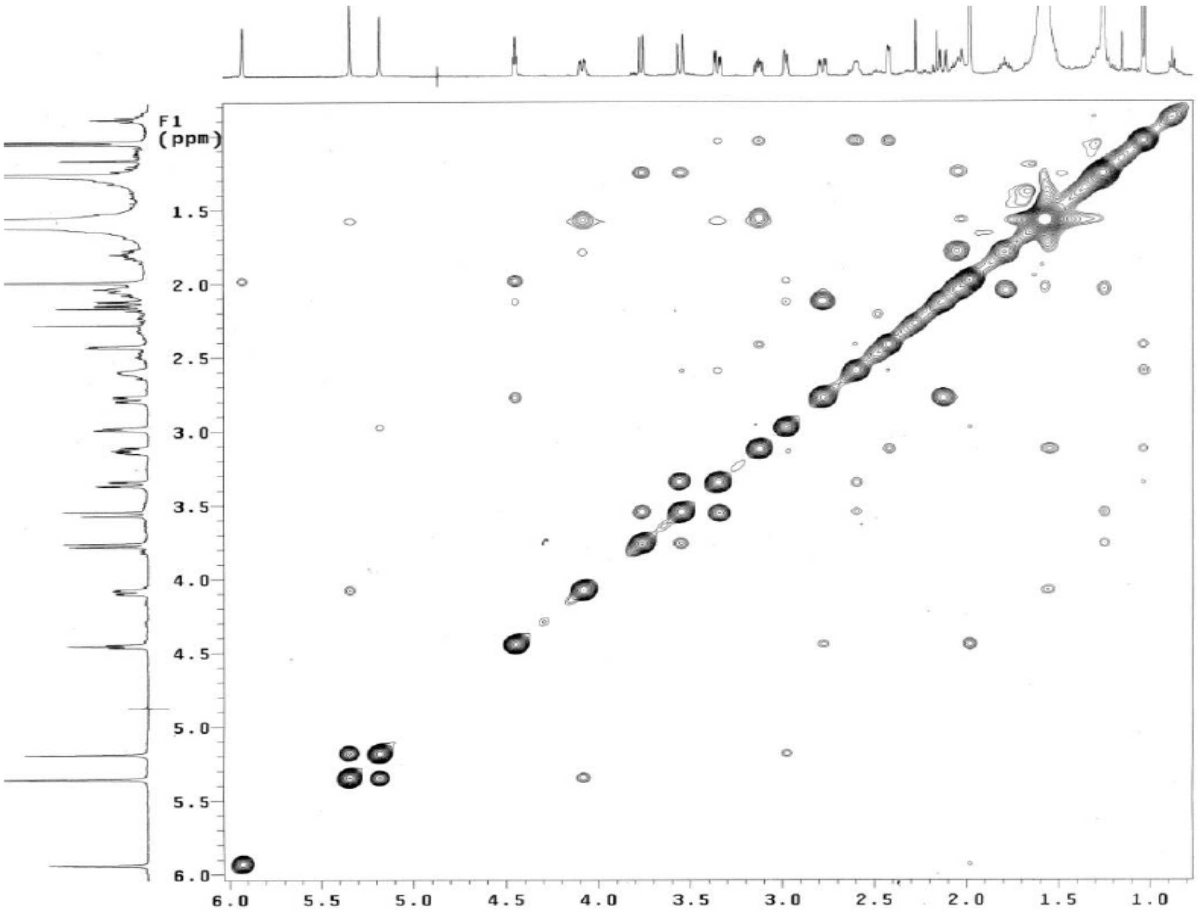

Figure S17. NOESY spectrum of **2** in $CDCl_3$

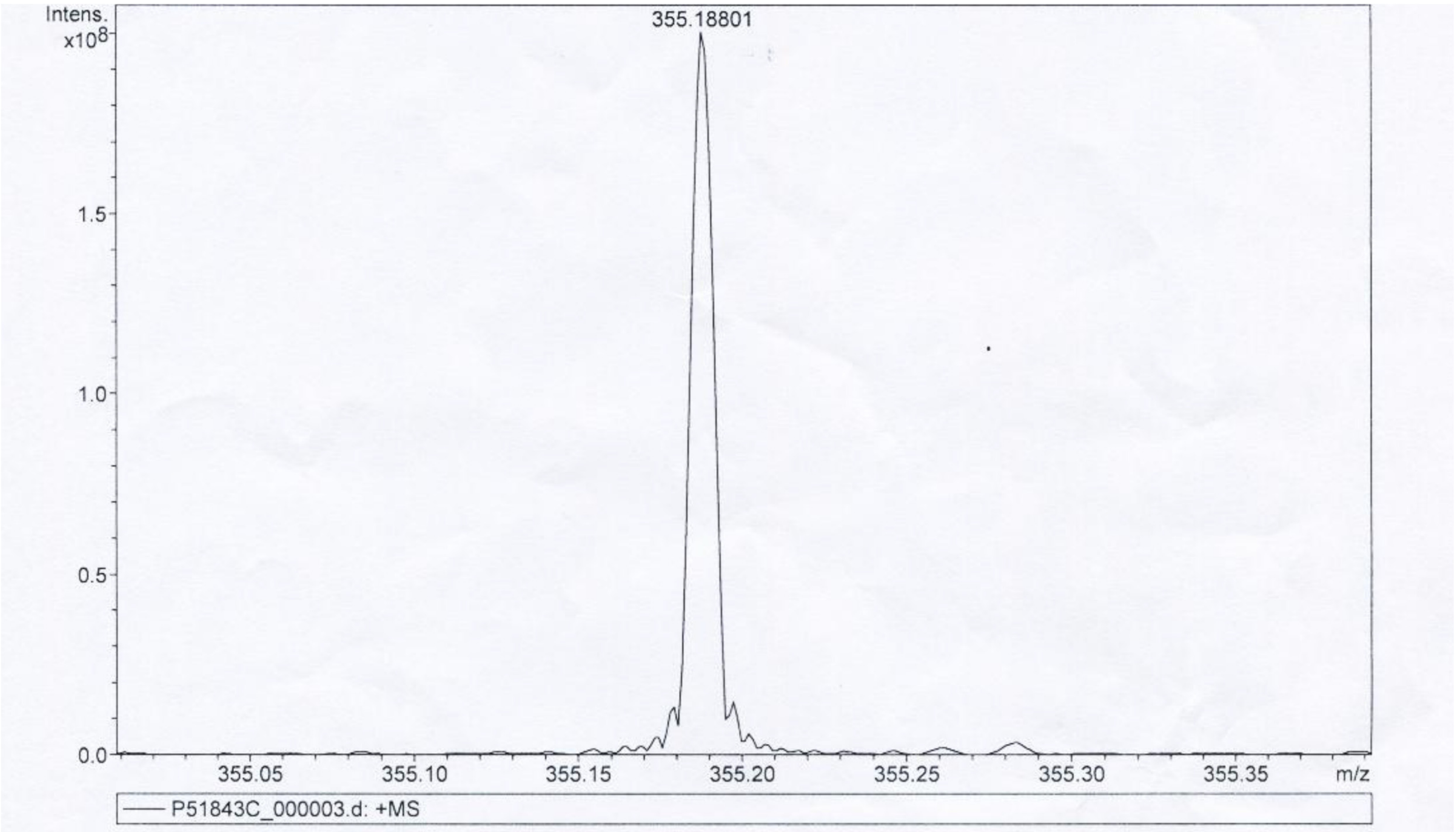

Meas. m/z	#	Formula	Score	m/z	err [mDa]	err [ppm]	mSigma	rdb	e⁻ Conf	N-Rule
355.18801	1	C 20 H 28 Na O 4	100.00	355.18798	-0.03	-0.09	6.9	6.5	even	ok

Figure S18. HRESMS spectrum of **3**

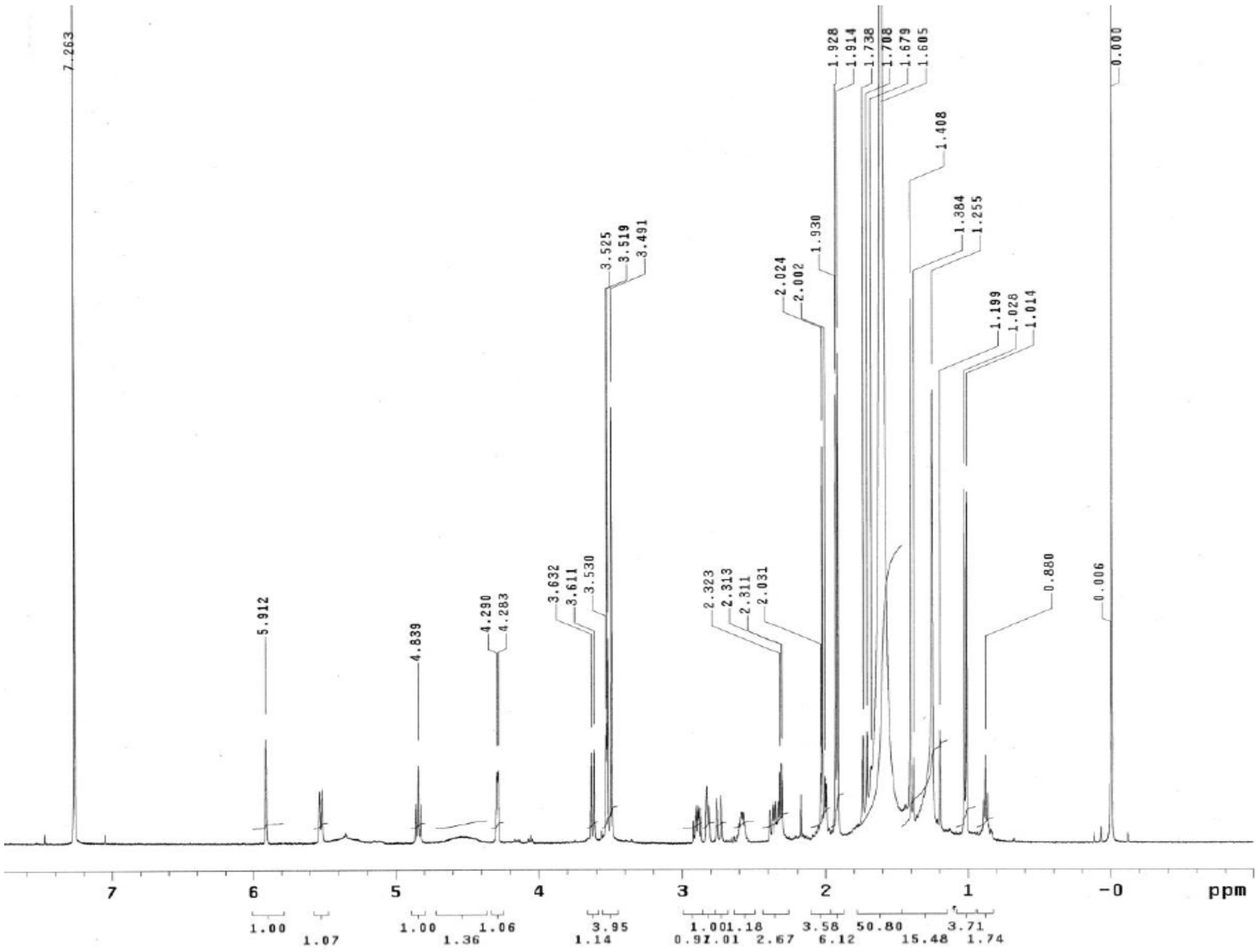

Figure S19. ^{1}H NMR spectrum of **3** in $CDCl_3$ at 500 MHz

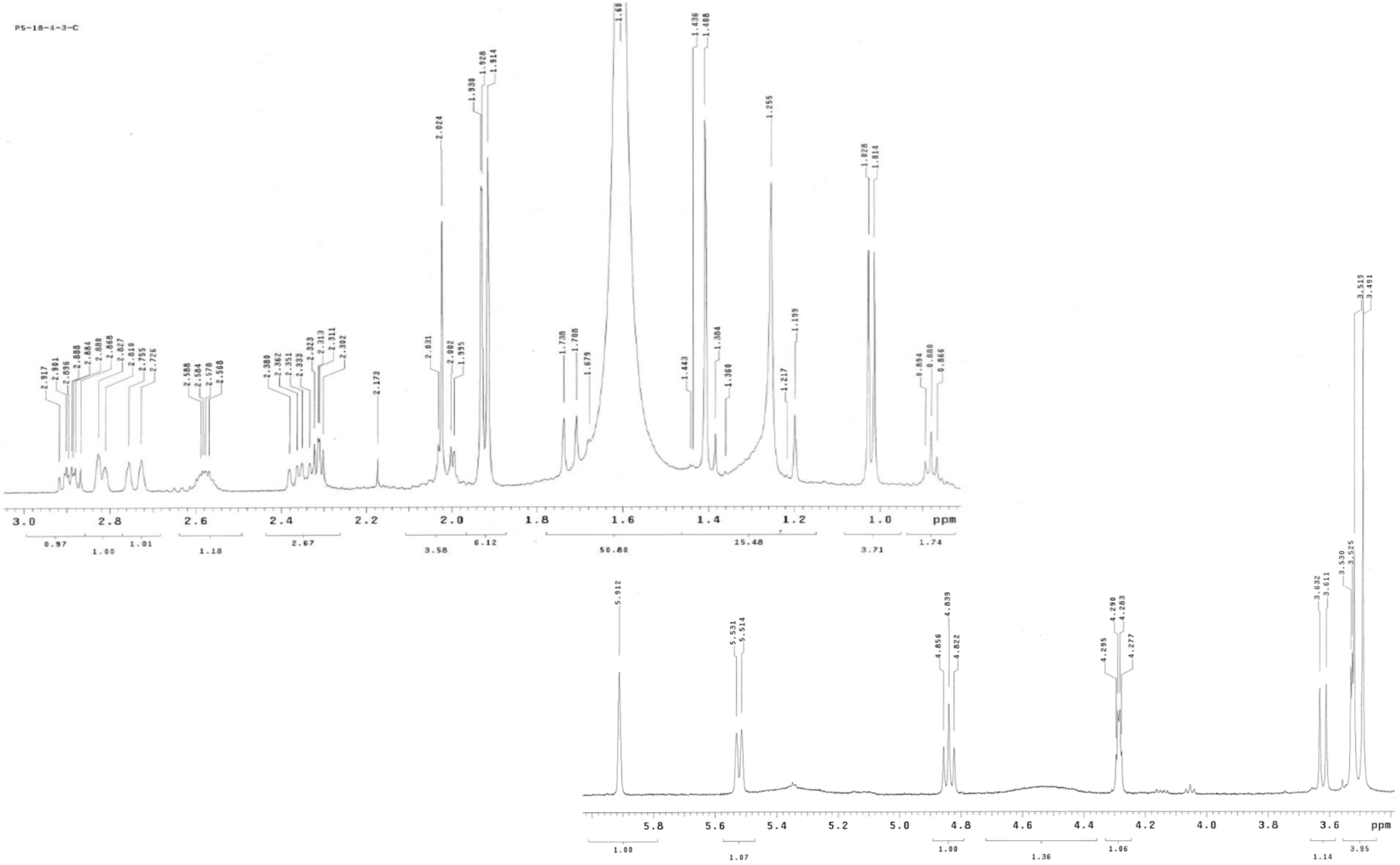

Figure S20. ^{1}H NMR spectrum of **3** in $CDCl_3$ (0.82~3.04 and 3.38~ 6.03 ppm) at 500 MHz

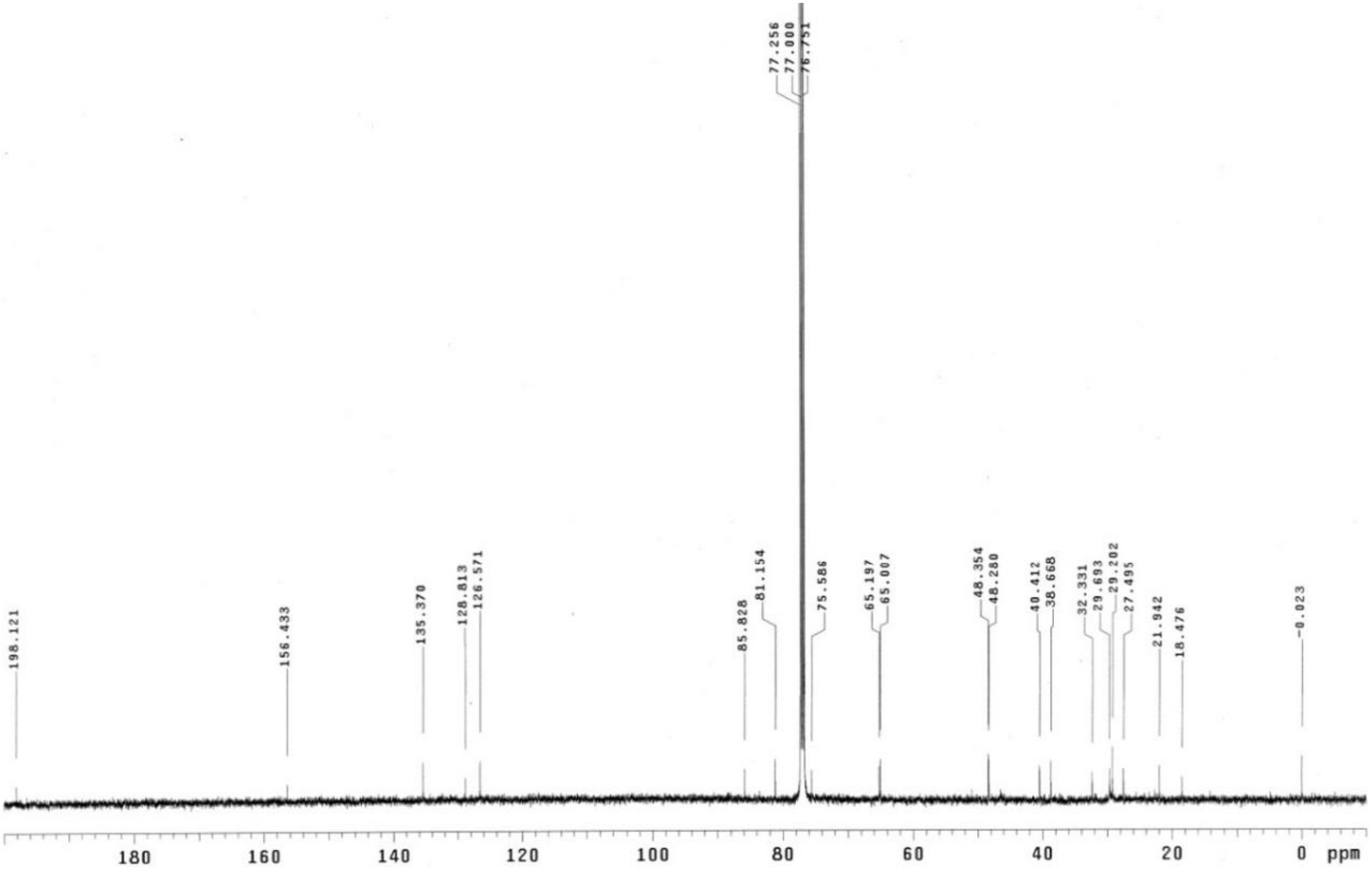

Figure S21. ^{13}C NMR spectrum of **3** in $CDCl_3$ at 125 MHz

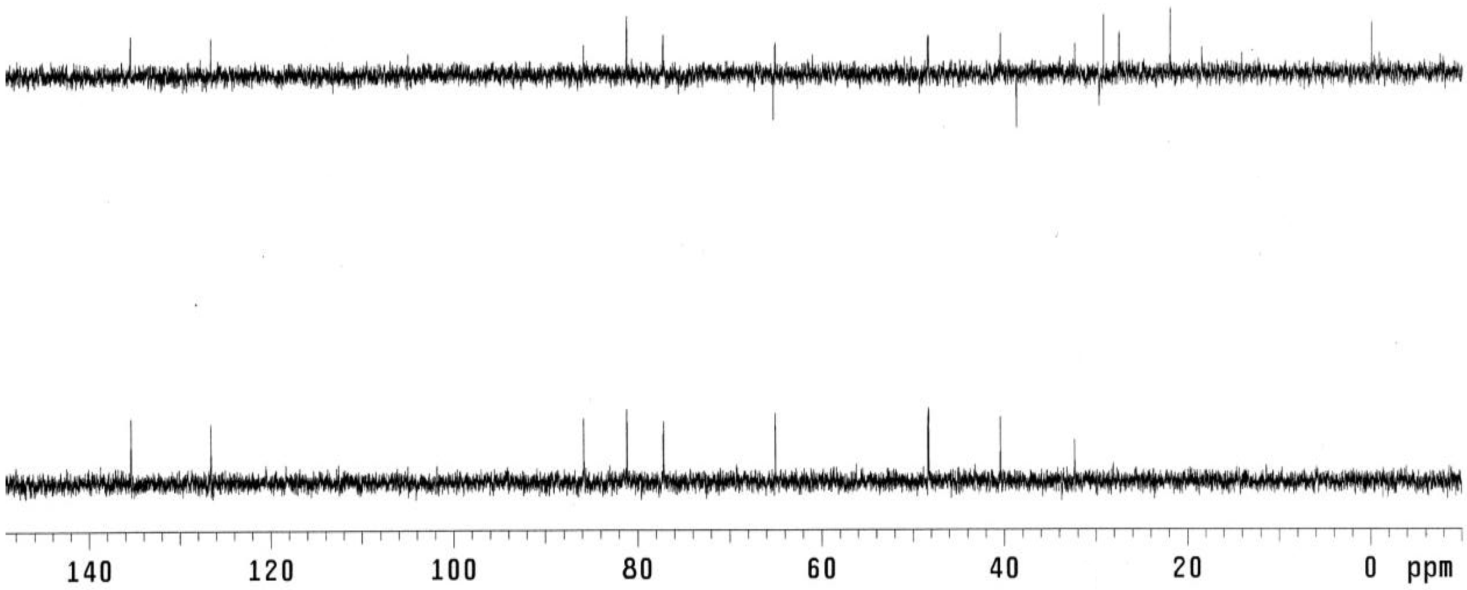

Figure S22. DEPT spectra of **3** in $CDCl_3$

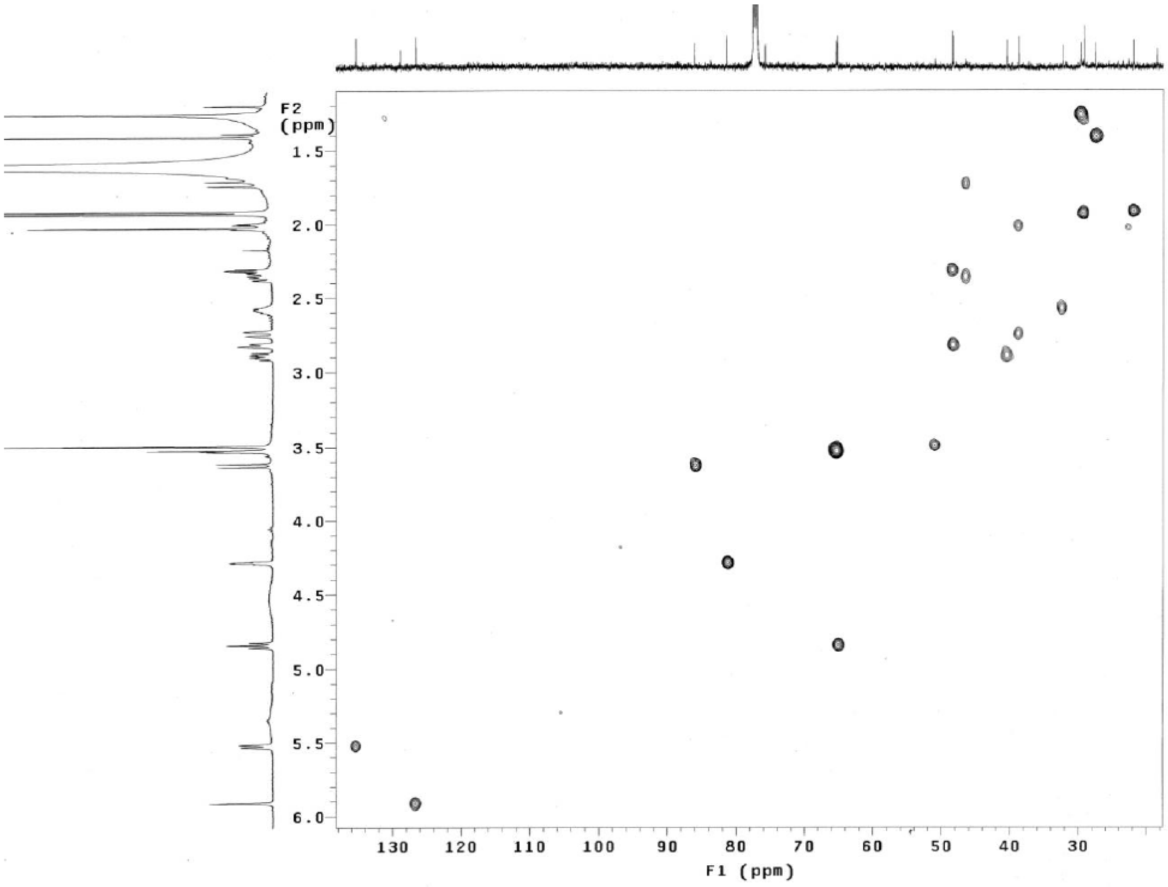

Figure S23. HSQC spectrum of **3** in $CDCl_3$

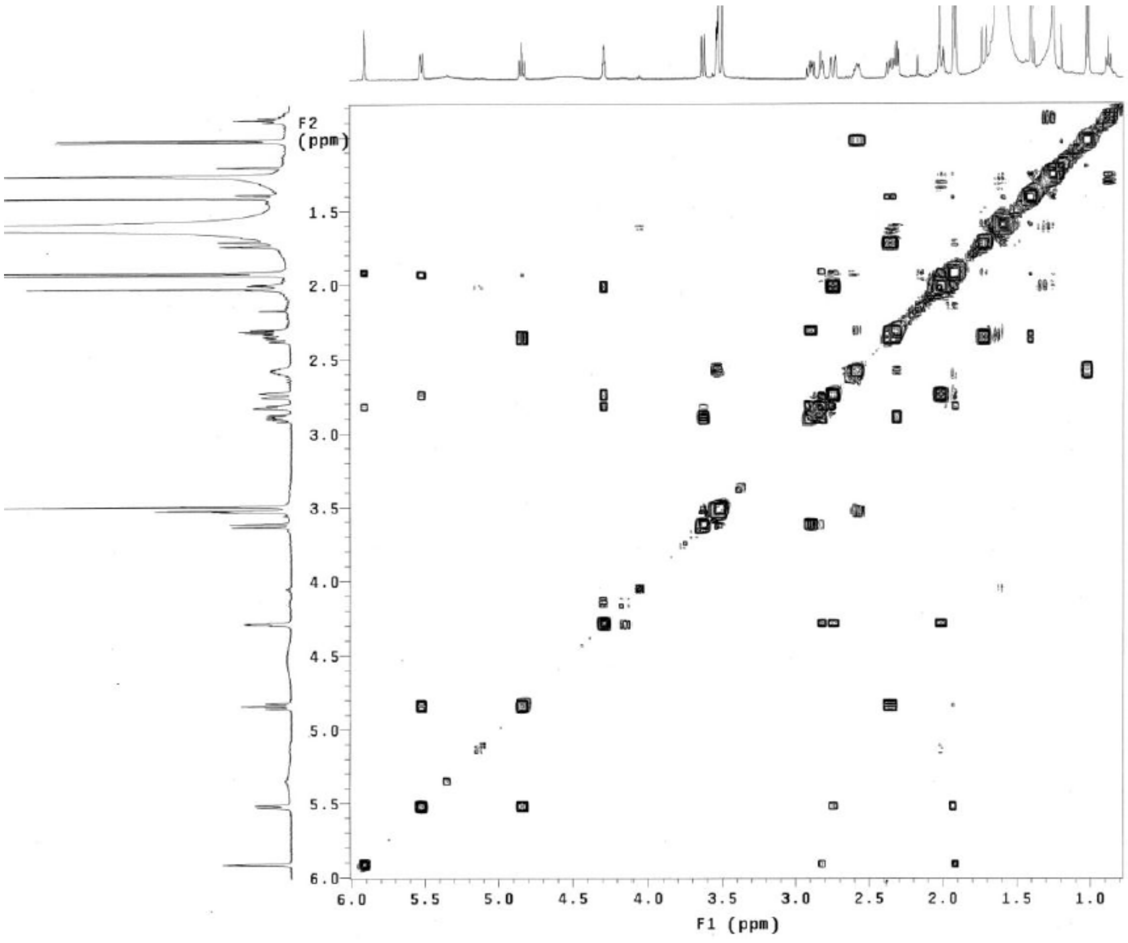

Figure S24. ^{1}H -^{1}H COSY spectrum of **3** in $CDCl_3$

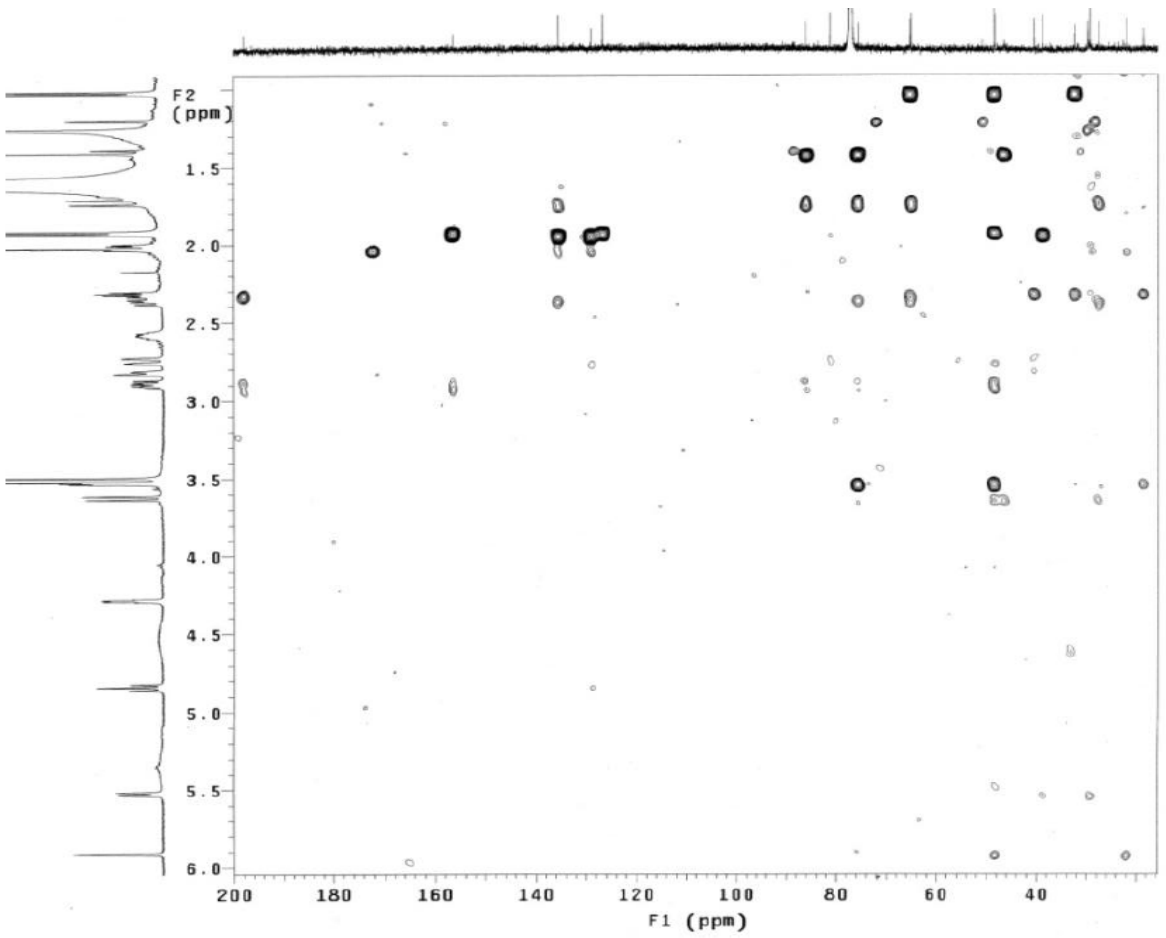

Figure S25. HMBC spectrum of **3** in $CDCl_3$

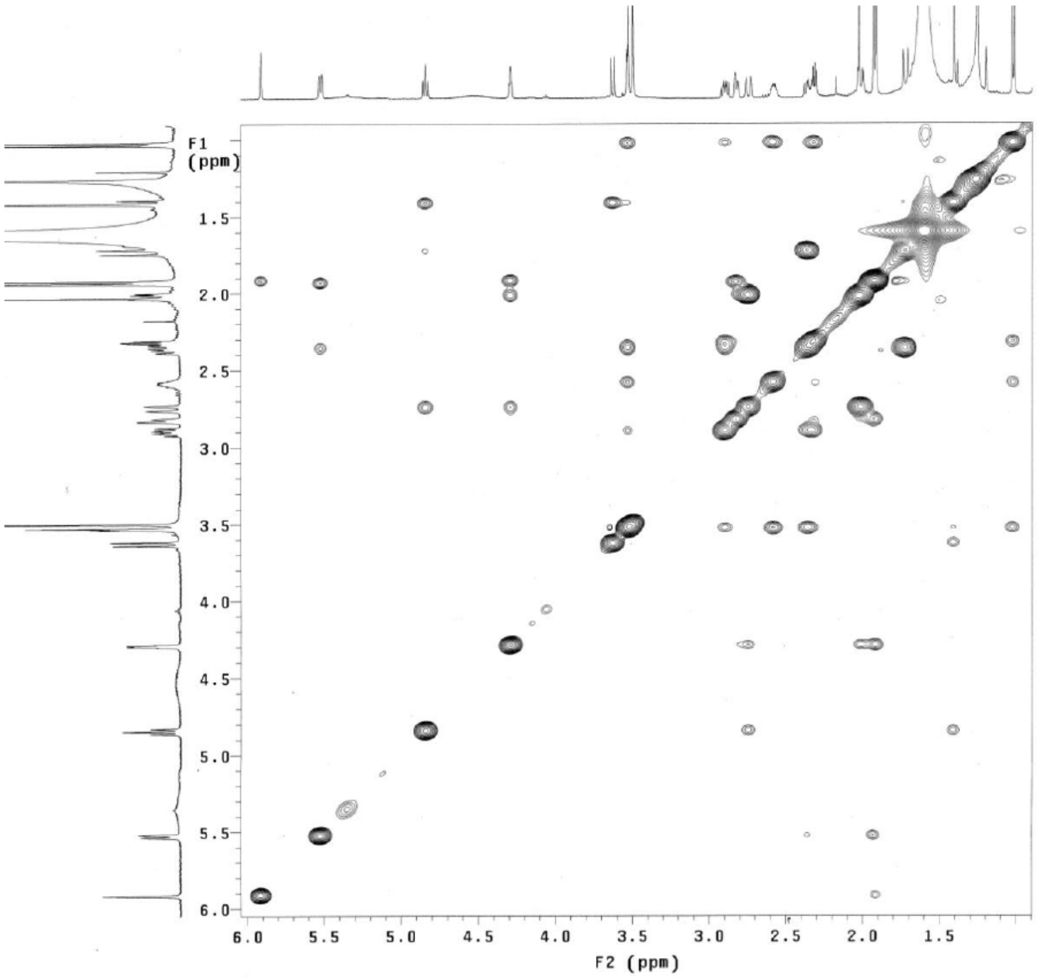

Figure S26. NOESY spectrum of **3** in $CDCl_3$

Table S1. Crystal data and structure refinement for **1**

Identification code	cu_1507121t2_0m_a	
Empirical formula	C22 H34 O7	
Formula weight	410.49	
Temperature	100(2) K	
Wavelength	1.54178 Å	
Crystal system	Orthorhombic	
Space group	P 21 21 21	
Unit cell dimensions	a = 8.3456(3) Å	= 90°.
	b = 10.6813(3) Å	= 90°.
	c = 23.4466(8) Å	= 90°.
Volume	2090.07(12) $Å^3$	
Z	4	
Density (calculated)	1.305 Mg/m^3	
Absorption coefficient	0.790 mm^{-1}	
F(000)	888	
Crystal size	0.20 x 0.18 x 0.17 mm^3	
Theta range for data collection	3.770 to 66.638°.	
Index ranges	-9<=h<=9, -12<=k<=9, -27<=l<=27	
Reflections collected	13326	
Independent reflections	3658 [R(int) = 0.0331]	
Completeness to theta = 67.679°	97.3 %	
Absorption correction	Semi-empirical from equivalents	
Max. and min. transmission	0.9492 and 0.7971	
Refinement method	Full-matrix least-squares on F^2	
Data / restraints / parameters	3658 / 0 / 268	
Goodness-of-fit on F^2	1.091	
Final R indices [I>2sigma(I)]	R1 = 0.0401, wR2 = 0.1064	
R indices (all data)	R1 = 0.0416, wR2 = 0.1075	
Absolute structure parameter	0.04(6)	
Extinction coefficient	n/a	
Largest diff. peak and hole	0.358 and -0.213 e.$Å^{-3}$	

Table S2. Atomic coordinates (x 104) and equivalent isotropic displacement parameters (Å2x 103) for **1**.

	x	y	z	U(eq)
C(13)	880(4)	1719(3)	5473(1)	22(1)
C(14)	310(4)	2439(3)	5994(1)	20(1)
C(15)	409(4)	1677(3)	6552(1)	22(1)
C(16)	2065(4)	1652(3)	6833(1)	20(1)
C(3)	3479(3)	3673(3)	6743(1)	16(1)
C(2)	2958(3)	3593(3)	6116(1)	16(1)
C(1)	1140(3)	3719(3)	6019(1)	17(1)
C(10)	1057(4)	4479(3)	5460(1)	18(1)
C(11)	1173(4)	3684(3)	4931(1)	21(1)
C(12)	1128(4)	2434(3)	4948(1)	22(1)
C(20)	1374(5)	4369(3)	4376(1)	28(1)
C(17)	-837(4)	2122(4)	6980(2)	28(1)
C(5)	4425(4)	5997(3)	6981(1)	18(1)
C(6)	4939(3)	6523(3)	6402(1)	17(1)
C(7)	3694(4)	7258(3)	6051(1)	17(1)
C(8)	2206(3)	6491(3)	5890(1)	17(1)
C(9)	2536(4)	5332(3)	5527(1)	17(1)
C(4)	3185(4)	4942(3)	7034(1)	16(1)
C(18)	5232(4)	3289(3)	6801(1)	20(1)
C(19)	3164(4)	8445(3)	6366(1)	20(1)
C(21)	7701(4)	7050(3)	6586(1)	22(1)
C(22)	8823(4)	8154(3)	6601(2)	27(1)
O(5)	1045(3)	579(2)	5484(1)	31(1)
O(6)	2485(3)	2831(2)	7077(1)	18(1)
O(1)	3721(3)	4527(2)	5778(1)	17(1)
O(2)	6195(2)	7455(2)	6513(1)	19(1)
O(3)	8094(3)	5974(2)	6626(1)	27(1)
O(4)	4528(2)	7602(2)	5537(1)	19(1)
O(7)	2334(3)	8644(2)	4808(1)	25(1)

U (eq) is defined as one third of the trace of the orthogonalized U^{ij} tensor.

Table S3. Bond lengths [Å] and angles [°] for **1**

C(13)-O(5)	1.225(4)	C(6)-O(2)	1.469(3)	C(17)-C(15)-C(16)	109.8(3)
C(13)-C(12)	1.464(5)	C(6)-C(7)	1.540(4)	C(17)-C(15)-C(14)	111.0(3)
C(13)-C(14)	1.520(4)	C(6)-H(6)	1.0000	C(16)-C(15)-C(14)	115.0(3)
C(14)-C(1)	1.534(4)	C(7)-O(4)	1.440(4)	C(17)-C(15)-H(15)	106.9
C(14)-C(15)	1.543(4)	C(7)-C(19)	1.533(4)	C(16)-C(15)-H(15)	106.9
C(14)-H(14)	1.0000	C(7)-C(8)	1.535(4)	C(14)-C(15)-H(15)	106.9
C(15)-C(17)	1.522(5)	C(8)-C(9)	1.527(4)	O(6)-C(16)-C(15)	112.2(2)
C(15)-C(16)	1.531(4)	C(8)-H(8A)	0.9900	O(6)-C(16)-H(16A)	109.2
C(15)-H(15)	1.0000	C(8)-H(8B)	0.9900	C(15)-C(16)-H(16A)	109.2
C(16)-O(6)	1.427(4)	C(9)-O(1)	1.437(3)	O(6)-C(16)-H(16B)	109.2
C(16)-H(16A)	0.9900	C(9)-H(9)	1.0000	C(15)-C(16)-H(16B)	109.2
C(16)-H(16B)	0.9900	C(4)-H(4A)	0.9900	H(16A)-C(16)-H(16B)	107.9
C(3)-O(6)	1.453(3)	C(4)-H(4B)	0.9900	O(6)-C(3)-C(18)	109.4(2)
C(3)-C(18)	1.526(4)	C(18)-H(18A)	0.9800	O(6)-C(3)-C(2)	108.7(2)
C(3)-C(2)	1.535(4)	C(18)-H(18B)	0.9800	C(18)-C(3)-C(2)	110.1(2)
C(3)-C(4)	1.538(4)	C(18)-H(18C)	0.9800	O(6)-C(3)-C(4)	102.4(2)
C(2)-O(1)	1.425(4)	C(19)-H(19A)	0.9800	C(18)-C(3)-C(4)	110.5(2)
C(2)-C(1)	1.540(4)	C(19)-H(19B)	0.9800	C(2)-C(3)-C(4)	115.4(2)
C(2)-H(2)	1.0000	C(19)-H(19C)	0.9800	O(1)-C(2)-C(3)	111.6(2)
C(1)-C(10)	1.542(4)	C(21)-O(3)	1.199(4)	O(1)-C(2)-C(1)	107.3(2)
C(1)-H(1)	1.0000	C(21)-O(2)	1.341(4)	C(3)-C(2)-C(1)	114.6(2)
C(10)-C(11)	1.507(4)	C(21)-C(22)	1.507(5)	O(1)-C(2)-H(2)	107.7
C(10)-C(9)	1.542(4)	C(22)-H(22A)	0.9800	C(3)-C(2)-H(2)	107.7
C(10)-H(10)	1.0000	C(22)-H(22B)	0.9800	C(1)-C(2)-H(2)	107.7
C(11)-C(12)	1.337(5)	C(22)-H(22C)	0.9800	C(14)-C(1)-C(2)	111.9(2)
C(11)-C(20)	1.502(4)	O(4)-H(4)	0.8400	C(14)-C(1)-C(10)	114.6(2)
C(12)-H(12)	0.9500	O(7)-H(7A)	0.9032	C(2)-C(1)-C(10)	102.4(2)
C(20)-H(20A)	0.9800	O(7)-H(7B)	0.9676	C(14)-C(1)-H(1)	109.2
C(20)-H(20B)	0.9800	O(5)-C(13)-C(12)	121.4(3)	C(2)-C(1)-H(1)	109.2
C(20)-H(20C)	0.9800	O(5)-C(13)-C(14)	121.4(3)	C(10)-C(1)-H(1)	109.2
C(17)-H(17A)	0.9800	C(12)-C(13)-C(14)	117.2(3)	C(11)-C(10)-C(9)	111.4(2)
C(17)-H(17B)	0.9800	C(13)-C(14)-C(1)	109.9(3)	C(11)-C(10)-C(1)	113.5(2)
C(17)-H(17C)	0.9800	C(13)-C(14)-C(15)	113.4(3)	C(9)-C(10)-C(1)	100.9(2)
C(5)-C(6)	1.530(4)	C(1)-C(14)-C(15)	114.4(2)	C(11)-C(10)-H(10)	110.2
C(5)-C(4)	1.534(4)	C(13)-C(14)-H(14)	106.1	C(9)-C(10)-H(10)	110.2
C(5)-H(5A)	0.9900	C(1)-C(14)-H(14)	106.1	C(1)-C(10)-H(10)	110.2
C(5)-H(5B)	0.9900	C(15)-C(14)-H(14)	106.1	C(12)-C(11)-C(20)	121.0(3)

Table S3. Bond lengths [Å] and angles [°] for **1** (contd.)

C(12)-C(11)-C(10)	122.5(3)	C(7)-C(8)-H(8A)	108.5	C(16)-O(6)-C(3)	118.1(2)
C(20)-C(11)-C(10)	116.5(3)	C(9)-C(8)-H(8B)	108.5	C(2)-O(1)-C(9)	109.8(2)
C(11)-C(12)-C(13)	123.3(3)	C(7)-C(8)-H(8B)	108.5	C(21)-O(2)-C(6)	118.3(2)
C(11)-C(12)-H(12)	118.3	H(8A)-C(8)-H(8B)	107.5	C(7)-O(4)-H(4)	109.5
C(13)-C(12)-H(12)	118.3	O(1)-C(9)-C(8)	112.4(2)	H(7A)-O(7)-H(7B)	102.6
C(11)-C(20)-H(20A)	109.5	O(1)-C(9)-C(10)	103.8(2)		
C(11)-C(20)-H(20B)	109.5	C(8)-C(9)-C(10)	113.0(2)		
H(20A)-C(20)-H(20B)	109.5	O(1)-C(9)-H(9)	109.2		
C(11)-C(20)-H(20C)	109.5	C(8)-C(9)-H(9)	109.2		
H(20A)-C(20)-H(20C)	109.5	C(10)-C(9)-H(9)	109.2		
H(20B)-C(20)-H(20C)	109.5	C(5)-C(4)-C(3)	120.2(3)		
C(15)-C(17)-H(17A)	109.5	C(5)-C(4)-H(4A)	107.3		
C(15)-C(17)-H(17B)	109.5	C(3)-C(4)-H(4A)	107.3		
H(17A)-C(17)-H(17B)	109.5	C(5)-C(4)-H(4B)	107.3		
C(15)-C(17)-H(17C)	109.5	C(3)-C(4)-H(4B)	107.3		
H(17A)-C(17)-H(17C)	109.5	H(4A)-C(4)-H(4B)	106.9		
H(17B)-C(17)-H(17C)	109.5	C(3)-C(18)-H(18A)	109.5		
C(6)-C(5)-C(4)	122.1(3)	C(3)-C(18)-H(18B)	109.5		
C(6)-C(5)-H(5A)	106.8	H(18A)-C(18)-H(18B)	109.5		
C(4)-C(5)-H(5A)	106.8	C(3)-C(18)-H(18C)	109.5		
C(6)-C(5)-H(5B)	106.8	H(18A)-C(18)-H(18C)	109.5		
C(4)-C(5)-H(5B)	106.8	H(18B)-C(18)-H(18C)	109.5		
H(5A)-C(5)-H(5B)	106.7	C(7)-C(19)-H(19A)	109.5		
O(2)-C(6)-C(5)	107.0(2)	C(7)-C(19)-H(19B)	109.5		
O(2)-C(6)-C(7)	103.4(2)	H(19A)-C(19)-H(19B)	109.5		
C(5)-C(6)-C(7)	118.1(2)	C(7)-C(19)-H(19C)	109.5		
O(2)-C(6)-H(6)	109.3	H(19A)-C(19)-H(19C)	109.5		
C(5)-C(6)-H(6)	109.3	H(19B)-C(19)-H(19C)	109.5		
C(7)-C(6)-H(6)	109.3	O(3)-C(21)-O(2)	125.2(3)		
O(4)-C(7)-C(19)	109.3(2)	O(3)-C(21)-C(22)	125.4(3)		
O(4)-C(7)-C(8)	108.8(2)	O(2)-C(21)-C(22)	109.5(3)		
C(19)-C(7)-C(8)	109.1(2)	C(21)-C(22)-H(22A)	109.5		
O(4)-C(7)-C(6)	104.5(2)	C(21)-C(22)-H(22B)	109.5		
C(19)-C(7)-C(6)	111.0(2)	H(22A)-C(22)-H(22B)	109.5		
C(8)-C(7)-C(6)	113.9(2)	C(21)-C(22)-H(22C)	109.5		
C(9)-C(8)-C(7)	115.1(2)	H(22A)-C(22)-H(22C)	109.5		
C(9)-C(8)-H(8A)	108.5	H(22B)-C(22)-H(22C)	109.5		
